# Supplementary material for: Towards a decision support tool for intensive care discharge: machine learning algorithm development using electronic healthcare data from MIMIC-III and Bristol, UK
Source: BMJ Open. 2019 Mar 7;9(3):e025925. doi: 10.1136/bmjopen-2018-025925 (PMC6429919; doi:10.1136/bmjopen-2018-025925)
Supplement: Author's manuscript [file bmjopen-2018-025925.draft_revisions.pdf]

BMJ Open

BMJ Open is committed to open peer review. As part of this commitment we make the peer review history of every article we publish publicly available.

When an article is published we post the peer reviewers' comments and the authors' responses online. We also post the versions of the paper that were used during peer review. These are the versions that the peer review comments apply to.

The versions of the paper that follow are the versions that were submitted during the peer review process. They are not the versions of record or the final published versions. They should not be cited or distributed as the published version of this manuscript.

BMJ Open is an open access journal and the full, final, typeset and author-corrected version of record of the manuscript is available on our site with no access controls, subscription charges or pay-per-view fees (http://bmjopen.bmj.com).

If you have any questions on BMJ Open's open peer review process please email info.bmjopen@bmj.com

BMJ Open

A machine learning approach to intensive care discharge.

Journal:	*BMJ Open*
Manuscript ID	bmjopen-2018-025925
Article Type:	Research
Date Submitted by the Author:	15-Aug-2018
Complete List of Authors:	McWilliams, Christopher; University of Bristol, Engineering Mathematics<br>Lawson, Daniel; University of Bristol, Integrative Epidemiology Unit, Population Health Sciences<br>Santos-Rodriguez, Raul; University of Bristol, Engineering Mathematics<br>Gilchrist, Iain; University of Bristol, Department of Experimental Psychology<br>Champneys, Alan; University of Bristol, Engineering Mathematics<br>Gould, Timothy; University Hospitals Bristol NHS Foundation Trust, Intensive Care Unit<br>Thomas, Mathew; University Hospitals Bristol NHS Foundation Trust, Intensive Care Unit<br>Bourdeaux, Christopher; University Hospitals Bristol NHS Foundation Trust, Intensive Care Unit
Keywords:	Adult intensive & critical care < ANAESTHETICS, Clinical audit < HEALTH SERVICES ADMINISTRATION & MANAGEMENT, Health informatics < BIOTECHNOLOGY & BIOINFORMATICS, Information technology < BIOTECHNOLOGY & BIOINFORMATICS

SCHOLARONE™
Manuscripts

A machine learning approach to intensive care discharge.

Christopher J. McWilliams□[1], Daniel J. Lawson[2], Raul Santos-Rodriguez[1], Iain D Gilchrist, Alan Champneys[1], Timothy H. Gould[4], Matthew J.C. Thomas[4], and Christopher P. Bourdeaux[4]

1 Department of Engineering Mathematics, University of Bristol
2 Integrative Epidemiology Unit, Population Health Sciences, University of Bristol
3 School of Experimental Psychology, University of Bristol
4 Intensive Care Unit, Queens Building, University Hospitals Bristol

*Corresponding author: chris.mcwilliams@bristol.ac.uk

Keywords: patient discharge, machine learning, clinical decision support, critical care, patient flow

Word count: 3554 (main text only)

For peer review only

Abstract

Objective
The primary objective is to work towards a clinical decision support tool that can improve discharge practice on the intensive care unit.

Design
We used two datasets of routinely collected patient data to test and improve upon a set of previously proposed discharge criteria.

Setting
Bristol Royal Infirmary general intensive care unit (GICU).

Patients
Two cohorts derived from historical datasets: 1933 intensive care patients from GICU in Bristol, and 10658 from MIMIC-III (a publicly available intensive care dataset).

Interventions
None.

Primary outcome measure
None

Results
In both cohorts few successfully discharged patients met the of all the discharge criteria. Both a random forest and a logistic classifier, trained on MIMIC and cross validated on GICU, demonstrated improved performance over the original criteria and generalised well between the cohorts. The classifiers showed good agreement on which features were most predictive of readiness-for-discharge, and these were generally consistent with clinical experience. By weighting the NLD criteria according to feature importance from the logistic model we showed improved performance over the original NLD criteria, while retaining good interpretability.

Conclusions
Our findings constitute a proof of concept for a decision support tool to run alongside a clinical information system, and streamline the process of discharge from the ICU.

For peer review only

Strengths and Limitations of this study:

- This study applies machine learning techniques to the problem of classifying patients that are ready for discharge from intensive care.
- Two cohorts of historical data are used, allowing cross-validation and a comparison of results between healthcare contexts.
- Our approach represents the first step towards a decision support tool that would help clinicians identify dischargeable patients as early as possible.

For peer review only

Introduction

Demand for intensive care unit (ICU) beds is rising at a time when resource is constrained[1]. In order to optimise the allocation of this resource, patients should be discharged from the ICU as soon as soon as they no longer require the specialist input provided there. The reduced ICU capacity caused by discharge delay can result in the delayed admission of emergency patients requiring ICU care[2,3]. Furthermore, patients remaining in the ICU after they are medically fit to leave experience detrimental effects on physical rehabilitation and psychosocial well-being[4].

The identification of individuals that are ready to leave ICU is a key component of patient flow through the hospital. At present this identification is a manual process, relying on physicians reviewing patients on a ward round at a standard point in time. There is a lack of formal guidance to inform discharge readiness and as such the process is sensitive to both the decision making heuristics of individual clinicians and structural factors within the hospital[5]. These issues may introduce undesirable variability in the timeliness of the discharge decision. A number of studies have looked to address this problem by attempting to standardise the discharge process[6]. One such study by Knight[7] proposed a set of physiological criteria to enable nurses to identify patients that are fit for discharge from a high dependency unit (HDU), thereby expediting the discharge process.

Increasingly ICUs are using clinical information systems (CIS) to collect, store and display physiological data. The availability of such routinely collected patient data presents the opportunity to apply methods from data science, with the potential to transform healthcare in a number of ways[8,9]. Two particular avenues for development are the automation of simple tasks[10] and the implementation of decision support systems[11], both of which would reduce the cognitive load of clinicians and free up scarce resource for tasks that require human expertise. We believe that the ICU discharge process is one area of healthcare practice that could be significantly improved by such data driven approaches.

In this study we investigated the possibility of using routinely collected data to identify patients that are ready for discharge. We studied two historical cohorts. One cohort consisted of patients treated on the general intensive care unit at the Bristol Royal Infirmary, while the second consisted of patients selected from the MIMIC-III database[12] (see Materials and Methods for details). Since there is no generally accepted definition of readiness-for-discharge, we adopted the criteria published by Knight[7] as a baseline for automation. Given the rational behind these criteria they represent a general and highly conservative set of constraints on physiology that characterise a patient as suitable for care on an acute ward (level 1 care). We initially posed the question: how many patients met the proposed criteria at the time they were declared ready for discharge? We then attempted to improve upon the performance of the original criteria by using machine learning techniques to identify patients that were ready for discharge. This work represents the first steps towards a decision support tool that will run in real time alongside our CIS and help clinicians identify dischargeable patients.

For peer review only

Methods

Discharge criteria

The nurse-led discharge (NLD) criteria proposed by Knight[7] consist of a set of constraints on various routinely collected vital signs and laboratory results. If a patient meets all the constraints for a period of at least four hours, Knight states that they may be safely discharged by a nurse. In order to test the NLD criteria on historical patient data we codified the constraints (see online supplementary file section A) into 15 binary tests, which are defined in table 1.

Test ID	Test name	Variable	Test condition
R0	Respiratory: airway	airway	airway patent
R1	Respiratory: FiO2	fio2	fio2 ≤ 0.6
R2	Respiratory: blood oxygen	spo2	spo2 ≥ 95 (%)
R3	Respiratory: bicarbonate	hco3	hco3 ≥ 19 (mmol/L)
R4	Respiratory: rate	resp (rate)	10 ≤ resp ≤ 30 (bpm)
C0	Cardiovascular: blood pressure	bp (systolic)	bp ≥ 100 (mmHg)
C1	Cardiovascular: heart rate	hr	60 ≤ hr ≤ 100 (bpm)
P	Pain	pain	0 ≤ pain ≤ 1
CNS	Central nervous system	gcs	gcs ≥ 14
T	Temperature	temp	36 ≤ temp ≤ 37.5 (C)
B0	Bloods: haemoglobin	haemoglobin	haemoglobin ≥ 9 (g/dL)
B1	Bloods: potassium	k	3.5 ≤ k ≤ 6.0 (mmol/L)
B2	Bloods: sodium	na	130 ≤ na ≤ 150 (mmol/L)
B3	Bloods: creatinine	creatinine	59 ≤ creatinine ≤ 104 (umol/L)
B4	Bloods: urea	bun	2.5 ≤ bun ≤ 7.8 (mmol/L)

Table 1: Codified version of the discharge criteria for application to electronic health record data. Here the fifteen criteria have been grouped into intuitive subsets and each assigned a test ID ('R0' to 'B4'). If all 15 criteria are met for a period of at least four hours the patient can be safely discharged.

For peer review only

Cohort selection

Subjects for this study were selected from two distinct historical data sources to form two patient cohorts. The inclusion criteria are detailed in section B of the online supplementary file. The first data source consists of the routinely collected data from 1933 patients treated on the general intensive care unit at the Bristol Royal Infirmary between 01/02/2015 and 01/02/2017. We refer to the cohort selected from this dataset as GICU. The second data source is derived from the MIMIC-III database[12], from which 10658 patients were selected to form the cohort referred to as MIMIC.

The use of two cohorts was motivated by two concerns. Firstly, the volume of data was significantly increased by the inclusion of the MIMIC cohort, increasing the volume of data available for training classifier algorithms. Secondly, the use of two cohorts allowed us to study the generalisation of our results between different patient populations under different healthcare systems.

Readiness-for-discharge

The key to testing and improving on discharge criteria was to be able to identify, from the historical data, patients that were ready-for-discharge (RFD) and not-ready-for-discharge (NRFD). These two subsets of patients, RFD and NRFD, define the positive and negative classes respectively. The datasets contain a callout for each patient, which marks the time at which a patient was declared clinically ready to leave the intensive care unit. For the positive class we selected patients at their time of callout who went on to have a positive outcome on leaving the unit. Conversely, patients who were declared clinically ready to leave the unit, but subsequently had a negative outcome, were included in the negative class. A positive outcome was defined as the patient leaving the hospital alive without readmission to ICU. A negative outcome was defined as either readmission to ICU during the same hospital admission, or in-hospital mortality after discharge from ICU. Given the low rates of negative outcome following callout in both MIMIC and GICU (see table 2), we generated further instances of the negative class, in order to balance the class sizes. To do this we sampled patients at between three and eight days prior to their callout (see supplementary section B: figures 1-3), under the assumption that patients were not-ready-for-discharge at this point in time, regardless of their eventual outcome state (positive or negative). Full details of this procedure are given in section B of the online supplementary file.

	patients	mortality	readmission	negative outcomes	mean(LOS)
MIMIC	10658	0.048	0.063	0.095	3.02
GICU	1933	0.038	0.031	0.062	4.92

Table 2: Summary of the two study cohorts: MIMIC and GICU. Negative outcome after discharge is defined as either readmission to ICU or patient mortality during the same hospital admission (not mutually exclusive). Length of stay(LOS) given in days.

Feature extraction

We used the same feature set to evaluate the NLD criteria and to train machine learning classifiers. We constructed either one or two features corresponding to each of the NLD criteria, depending on the criteria in question and on data availability. For example, the features 'resp min' and 'resp max' were used to test the criterion R4, whereas the single feature 'bun' was used to test B4. Where possible the feature values were calculated from a four hour sample window, as specified by the original NLD criteria. In the cases where no data was available during the four hour window, an extended 36 hour window was used. This extended window was mainly relevant for infrequently measured laboratory test results (see table 1 in section C of the online supplementary file). Full details and justification of the feature extraction procedure are provided in section C of the online supplementary file, and the resulting 18 features are listed in the first column of table 3.

The results presented in the main text represent a complete case analysis, with all instances containing missing data entries removed. This removal reduced the sizes of the MIMIC and GICU feature matrices to 5033 and 1858 instances respectively. The validity of the complete case analysis was investigated with a sensitivity analysis that used k-nearest neighbour imputation[13] to fill missing data (see section D of the online supplementary file). When training and testing the machine learning classifiers, features were standardised by subtracting the column mean and dividing by the standard deviation. The complete case feature matrices are visualised in figure 1 using the t-SNE algorithm[14] (see supplementary section D: figure 4 for the equivalent imputed feature matrices).

For peer review only

Analysis of NLD criteria

Knight originally specified that all 15 criteria must be met in order to allow safe discharge by a nurse[7]. Following this specification we evaluated the criteria for both MIMIC and GICU, determining which instances were classified as RFD and NRFD, and comparing these results to ground-truth. We then further investigated the performance of the NLD criteria as a classification system, by relaxing the constraint that all 15 tests must be passed in order to make an RFD classification. Instead we used the NLD criteria to produce probability estimates of being RFD, by summing the number of tests passed and dividing by 15 to produce a normalised output between 0 and 1. In this formulation each of the 15 criteria contribute equally to the RFD probability. Using the probability outputs it was possible to evaluate the performance of the NLD criteria in the same way as the machine learning classifiers described below.

Machine learning classifiers

To improve upon the performance of the NLD criteria, we trained and tested two machine learning classifiers: a random forest (RF)[15], and a logistic classifier (LC)[16]. These two algorithms were chosen for their simplicity in implementation and ease of interpretation in their predictive output. Both classifiers were optimised over a range of hyper-parameter values using cross-validation (see section E of the online supplementary file). To do so, we produced an ensemble of 100 randomised train:test data splits for both MIMIC and GICU (70:30 and 67:33 respectively). We then optimised each classifier using cross-validation, with the larger subset of MIMIC as training data and the larger subset of GICU as validation data. Having obtained the optimised classifier for the given data split, the classifier was then refitted to the full training set (MIMIC + GICU). The remaining data (i.e. the smaller subsets of MIMIC and GICU) were then used to test the performance of the optimised classifier. This optimisation/testing procedure was repeated for all 100 data splits in the ensemble to produce estimates of the mean and standard deviation of the classifier performance. The methodology described was intended to produce classifiers that generalised well from MIMIC to GICU, and by extension to other patient populations.

Both the RF and LR classifiers contained mechanisms to promote model sparsity. For the RF this mechanism was a restriction in the hyper-parameters *tree_depth* and *max_features* (see section E of the online supplementary file), while for the LC this was ‘l1’ -regularisation. In both cases the intention was to learn which features were most and least predictive of readiness-for-discharge. For the RF, feature importance was given by the total reduction in Gini impurity provided by each feature. Intuitively this metric captures the reduction in uncertainty brought about a given feature, therefore giving a measure of how important that feature is, on average, in classification decisions. For the LC, the importance was given by the absolute value of the coefficient for each feature in the trained model, capturing the effect of a small change in a given feature value on the prediction probability with all other feature values held constant.

Classifier performance was evaluated across a range of prediction thresholds by producing receiver-operator-characteristic (ROC) and precision-recall (PRC) curves[17]. Given the need to minimise the false positive rate, while retaining high recall, we chose to use the partial area under the ROC curve (pAUC) as the overall performance metric[18]. The pAUC was evaluated up to a false positive rate of 0.3, using linear interpolation to approximate the true positive rate at this point on the ROC curve. Performance was evaluated in this way for the RF and LR classifiers, and for the original NLD criteria.

For peer review only - http://bmjopen.bmj.com/site/about/guidelines.xhtml

Results.

The original specification of the NLD criteria proved to be highly conservative as expected, producing low false positive and true positive rates for both cohorts (supplementary section D: tables 2-5). The true positive rates for MIMIC and GICU were 0.4% and 6.4% respectively. As such the NLD criteria were sufficiently insensitive as to call into question their usefulness in their current form.

By relaxing the constraint that all 15 tests must be passed, the NLD criteria were able to successfully identify more patients as RFD. This is illustrated in figure 2 for a single train:test data split, alongside the performance of the corresponding optimal random forest classifier (see also supplementary section D: figure 5). On this data split the NLD criteria obtained precisions of ~0.8 at a recall of 0.4 for both cohorts. The performance gain obtained by using a random forest was significant, with precisions of >0.8 at a recall of 0.7 for both cohorts. At a false positive rate of 3.6% the NLD criteria produced 66 true positives, while the random forest produced 113.

Feature	Importance (RF)	Importance (LC)	Rank (RF)	Rank (LC)
gcs_min	0.352 (±0.054)	1.127 (±0.050)	0	0
airway	0.302 (±0.048)	0.870 (±0.038)	1	1
bun	0.034 (±0.007)	0.437 (±0.047)	4	2
fio2	0.048 (±0.007)	0.317 (±0.024)	2	3
temp_max	0.024 (±0.008)	0.263 (±0.126)	7	4
haemoglobin	0.029 (±0.006)	0.256 (±0.029)	5	5
resp_max	0.017 (±0.004)	0.236 (±0.038)	10	6
resp_min	0.045 (±0.015)	0.233 (±0.053)	3	7
temp_min	0.014 (±0.004)	0.213 (±0.126)	12	8
hr_min	0.022 (±0.005)	0.181 (±0.044)	8	9
hr_max	0.025 (±0.005)	0.168 (±0.044)	6	10
spo2_min	0.012 (±0.004)	0.158 (±0.027)	14	11
na	0.009 (±0.003)	0.110 (±0.032)	16	12
bp_min	0.012 (±0.004)	0.059 (±0.027)	13	13
hco3	0.010 (±0.004)	0.051 (±0.028)	15	14
k	0.008 (±0.003)	0.041 (±0.022)	17	15
creatinine	0.021 (±0.005)	0.031 (±0.027)	9	16
pain	0.016 (±0.008)	0.021 (±0.018)	11	17

Table 3: Feature importances given by the random forest (RF) and logistic classifier (LC), evaluated over 100 train:test data splits. Importance values are given as: mean(±standard deviation). Features are ranked according to mean importance value, and the table is ordered according to the ranking given by the logistic classifier.

Broadly the two classifiers agreed as to which features were most predictive of readiness-for-discharge (see table 3). Eight of the logistic classifier's top ten important features were also ranked

For peer review only

in the top ten by the random forest, when averaged over the ensemble of 100 data splits. The Spearman's rank correlation coefficient between the feature rankings was 0.800 (p=0.00006), and both classifiers ranked *gcs_min* and *airway* as the two most important features by a significant margin. The inclusion of instances with missing data did little to change these feature rankings (see supplementary section D: figures 6-7).

Figure 3 summarises classifier performance, quantified using the partial area under the ROC curve (pAUC), over the ensemble of data splits. On average the original NLD criteria performed slightly better for GICU than MIMIC. The two machine learning classifiers performed similarly well, producing large gains in pAUC over the NLD for both cohorts, and higher pAUC values for MIMIC than GICU. There was little to distinguish between the random forest and logistic classifiers based on pAUC. In the sensitivity analysis (see supplementary section D: figure 8 and table 6) both machine learning classifiers still performed better than the NLD criteria, but all classifiers showed a performance drop for MIMIC and one classifier tended to perform better for each cohort (the random forest and logistic classifiers for MIMIC and GICU respectively).

Given the similarity in classifier performances we chose to use the average feature importances of the simpler model – the logistic classifier - to weight the NLD criteria. The weighted version of the NLD criteria (referred to as NLD_{opt}) performed better than the original criteria when tested on both MIMIC and GICU. On MIMIC the performance gain was larger, with pAUC scores approaching those of the machine learning classifiers. Qualitatively the same effect was observed under the sensitivity analysis.

For peer review only

Examples in practice

To illustrate the results in a more human-interpretable fashion we have selected five informative examples from the GICU cohort. Table 4 summarises the performance of the different classification systems for these five examples, which are labelled as true or false positive/negative (TP,FP,TN,FN) according to how they would be classified under the original nurse-led discharge criteria. One patient (ID 4065) is included twice: once at 72 hours before callout, and again at the time of callout. All four classification systems show an increased RFD probability for this patient between the two time points, as would be expected. Patient 868 is a false negative under the original criteria - despite failing two criteria (C0 and T) their callout was successful. The three alternative classification systems (NLD_{opt} , RF and LC) correctly assign a high RFD probability for this patient, therefore improving upon the original criteria.

For these select examples the logistic classifier is the only system to assign lower RFD probabilities to the two false positive instances (1034 and 10783) than to the true positive instance (4065). Despite this correct ordering by the logistic classifier its RFD probabilities for the false positives are relatively high. Given the conservative nature of the NLD criteria it is expected that correct classification of the false positive instances is a hard problem.

Patient	NLD	NLD_{opt}	RF	LC	NLD fails	Notes
1034 (FP)	0.010 (1.0)	0.010 (1.0)	0.071 (0.784)	0.096 (0.765)	-	Patient admitted to ICU post surgery (primary lung tumour). Discharge to ward. Readmitted within 24 hours with bacterial pneumonia.
10783 (FP)	0.010 (1.0)	0.010 (1.0)	0.035 (0.819)	0.163 (0.716)	-	Patient admitted to ICU with secondary hepatic tumour. Appears to be RFD at 96 hours prior to callout.
4065 (TN)	1.0 (0.467)	0.464 (0.702)	0.368 (0.494)	0.395 (0.450)	R2, R4, C0, P, B1, B3, B4	Patient admitted to ICU with intracranial abscess. Not ready for discharge at 72 hours prior to callout.
4065 (TP)	0.010 (1.0)	0.010 (1.0)	0.077 (0.780)	0.047 (0.812)	-	Same patient as above. RFD at time of callout.
868 (FN)	0.113 (0.867)	0.046 (0.939)	0.080 (0.777)	0.054 (0.806)	C0, T	Patient admitted with malignant large bowel tumour. Appears NRFD at time of callout. Positive outcome.

Table 4: Example patients and their scores given by the four classification systems: the original nurse led discharge criteria (NLD); the weighted criteria(NLD_{opt}); the random forest (RF); and the logistic classifier (LC). The reporting score given is the false positive rate at the point where the patient falls on the ROC curve, such that a lower score indicates a higher probability of being RFD according to the given classifier (explicitly, the reporting metric gives the number of false positives that must be accepted before this patient can be classified RFD). These scores can be compared across classifiers. The raw (non-calibrated) classifier scores are given in brackets and cannot be compared across classifiers. The results FP,TN,TP, and FN indicated in the first column correspond to the outcomes of the original NLD criteria. The column 'NLD fails' specifies, where relevant, which of the NLD criteria were not met (criteria IDs correspond to those in table 1).

For peer review only

Discussion

Identifying which patients are suitable for ICU discharge is complex[1]. Delayed and out of hours discharges are associated with an increased mortality[19], and patients in ICU who could be managed on the ward put an increasing strain on resources. The determination of ready-for-discharge status is influenced by many unmeasured factors such as ICU census[20] and this leads to unwarranted variation in clinical decision making. The decision to declare someone fit for discharge is based on the judgement of individual clinicians and is likely to be given a lower priority than decisions around treatment options for patients that are more unwell in the ICU.

In this study we have used routinely collected data to test a set of discharge criteria[7] against two machine learning classifiers. The discharge criteria were found to be highly conservative, with very few successfully discharged patients meeting all the required criteria. This low sensitivity was expected since the original criteria were designed to be implemented independently of usual ward rounds, and false positives in this scenario could have serious consequences. A random forest and a logistic classifier both performed better than the clinically derived discharge criteria when trained on the same feature set. The two classifiers broadly agreed on which features were most predictive of readiness-for-discharge. Weighting the original discharge criteria with the features importances of the logistic classifier improved their classification performance.

An important novel aspect of this work is the use of MIMIC-III to increase the volume of training data available locally. Such applications of machine learning techniques to datasets that span institutions and healthcare settings will be of increasing value as more intensive care datasets become available for research[21]. Our results demonstrate the feasibility of using combined datasets in this way to derive clinical insight, and could be developed by the application of transfer learning approaches[22] to characterise systematic differences between data distributions.

The features identified as important by the classifiers were clinically meaningful. Clinicians will recognise that coma score, respiratory function and renal function are strongly related to successful ICU discharge. It is perhaps surprising that cardiovascular parameters were not ranked higher. We propose two possible mechanisms to account for this apparent discrepancy. Firstly, it may be a consequence of patient heterogeneity on the general intensive care unit[23]. For example, cardiovascular parameters may be highly predictive for cardiac patients yet much of this predictive power is lost in our attempt to fit a general model for the whole ICU population. Secondly, it may be a due to our simplistic choice of features, which use the absolute values of physiological parameters. For some parameters we suggest that other features such as the trend, variance, or change since time of admission may be more predictive. For example, improvement in blood pressure may be more informative than absolute blood pressure.

Our feature set was chosen to be directly analogous with those used by Knight's criteria, to allow a direct comparison in performance. This feature set is somewhat restrictive, having been originally designed to be manually recorded by nurses using paper charts. The rich wealth of data held in electronic charting systems could be better exploited by including more physiological parameters, and engineering more predictive features. In particular our modelling did not make use of demographic information, diagnoses, comorbidities or interventions. The later is of particular importance since many of patient's physiological parameters are controlled by clinical intervention during their stay in ICU. For example, a patient on vassopressors may have close to normal blood pressure despite suffering form server cardiovascular complications. Therefore, conditioning features on medical interventions represents one avenue to significantly boost performance. Methods to account for patient heterogeneity and individual disease trajectories would also be worth investigating[23,24]. Although the inclusion of entries with missing data did not qualitatively alter the results of our complete case analysis, the development of a robust imputation

For peer review only - http://bmjopen.bmj.com/site/about/guidelines.xhtml

strategy would improve performance by making best use of the available training data and exploiting the value in missingness[25]▪.

The aggregate effects of the improvement gains produced by our machine learning approach could be beneficial to many[26]▪. Therefore, we suggest that a future decision support tool embedded within a CIS should use these techniques to alert clinicians when patients appear fit for discharge. The increasing worldwide adoption of information systems in intensive care would make such a system widely applicable in years to come[27]▪. We have shown in previous work that subtle changes to the presentation of information can have significant impact on clinical decision making[28]▪. Therefore we anticipate such a tool has the potential to significantly streamline the discharge process. Two issues would need to be addressed prior to implementation on the ICU. The first is the human-interpretability of the classifier output. Depending on the machine learning approach a number of solutions exist[29]▪, including the approximation of random forests with simple decision trees[30]▪▪, that would allow clinicians to engage with the reasons behind a given classification. The second is the ambiguity behind the ground-truth used to train the classifiers. For example, some patients in the datasets used did not have a recorded callout status and some patients may have been ready for discharge prior to callout. A live implementation with a human-in-the-loop[31]▪ could use clinician input to update ground-truth in such situations and improve learning.

For peer review only

Conclusion

We have shown that it is possible to apply machine learning techniques to routinely collected ICU data in order to solve a significant clinical and operational problem. This approach offers promise in a number of areas. We plan to focus on the development and deployment of a decision support tool in order to inform clinicians of patients that could potentially be discharged from ICU, in order to streamline the process and reduce unnecessary ICU stay. As more patient data becomes available in the wider hospital setting, there is extensive scope to use such data-driven methods to solve the problem of poor patient flow through hospitals.

For peer review only

References

1 Rubenfeld GD, Rhodes A. How many intensive care beds are enough? *Intensive Care Med* 2014;**40**:451–2. doi:10.1007/s00134-014-3215-x

2 Chalfin DB, Trzeciak S, Likourezos A, *et al.* Impact of delayed transfer of critically ill patients from the emergency department to the intensive care unit. *Crit Care Med* Published Online First: 2007. doi:10.1097/01.CCM.0000266585.74905.5A

3 Cardoso LT, Grion CM, Matsuo T, *et al.* Impact of delayed admission to intensive care units on mortality of critically ill patients: a cohort study. *Crit Care* 2011;**15**:R28. doi:10.1186/cc9975

4 Howell MD. Managing ICU throughput and understanding ICU census. *Curr Opin Crit Care* 2011;**17**:626–33. doi:10.1097/MCC.0b013e32834b3e6e

5 Capuzzo M, Moreno RP, Alvisi R. Admission and discharge of critically ill patients. *Curr Opin Crit Care* 2010;**16**:499–504. doi:10.1097/MCC.0b013e32833cb874

6 Stelfox HT, Lane D, Boyd JM, *et al.* A Scoping Review of Patient Discharge From Intensive Care. *Chest* 2015;**147**:317–27. doi:10.1378/chest.13-2965

7 Knight G. Nurse-led discharge from high dependency unit. *Nurs Crit Care*;**8**:56–61.

8 Obermeyer Z, Lee TH. Lost in Thought — The Limits of the Human Mind and the Future of Medicine. *N Engl J Med* 2017;**377**:1209–11. doi:10.1056/NEJMp1705348

9 Docherty AB, Lone NI. Exploiting big data for critical care research. *Curr Opin Crit Care* 2015;**21**:467–72. doi:10.1097/MCC.0000000000000228

10 Sohn E, Roski J, Escaravage S, *et al.* Four Lessons In The Adoption Of Machine Learning In Health Care. *Heal Aff Blog* 2017. doi:10.1377/hblog20170509.059985

11 Kawamoto K, Houlihan CA, Balas EA, *et al.* Improving clinical practice using clinical decision support systems: a systematic review of trials to identify features critical to success. *BMJ* 2005;**330**:765. doi:10.1136/bmj.38398.500764.8F

12 Johnson AEW, Pollard TJ, Shen L, *et al.* MIMIC-III, a freely accessible critical care database. *Sci Data* 2016;**3**:160035. doi:10.1038/sdata.2016.35

13 Batista GEAPA, Monard MC. An analysis of four missing data treatment methods for supervised learning. *Appl Artif Intell* 2003;**17**:519–33. doi:10.1080/713827181

14 Maaten L van der, Hinton G. Visualizing Data using t-SNE. *J Mach Learn Res* 2008;**9**:2579–605.

15 Liaw A, Wiener M. Classification and Regression by RandomForest. *R News* 2002;**2**:18–22.

16 Dreiseitl S, Ohno-Machado L. Logistic regression and artificial neural network classification models: a methodology review. *J Biomed Inform*;**35**:352–9.

For peer review only

17 Davis J, Goadrich M. The relationship between Precision-Recall and ROC curves. In: *Proceedings of the 23rd international conference on Machine learning - ICML '06*. New York, New York, USA: : ACM Press 2006. 233–40. doi:10.1145/1143844.1143874

18 Zou KH, O'Malley AJ, Mauri L. Receiver-Operating Characteristic Analysis for Evaluating Diagnostic Tests and Predictive Models. *Circulation* 2007;**115**:654–7. doi:10.1161/CIRCULATIONAHA.105.594929

19 Goldfrad C, Rowan K. Consequences of discharges from intensive care at night. *Lancet (London, England)* 2000;**355**:1138–42. doi:10.1016/S0140-6736(00)02062-6

20 Lin F, Chaboyer W, Wallis M. A literature review of organisational, individual and teamwork factors contributing to the ICU discharge process. *Aust Crit Care* 2009;**22**:29–43. doi:10.1016/j.aucc.2008.11.001

21 Harris S, Shi S, Brealey D, *et al.* Critical Care Health Informatics Collaborative (CCHIC): Data, tools and methods for reproducible research: A multi-centre UK intensive care database. *Int J Med Inform* 2018;**112**:82–9. doi:10.1016/j.ijmedinf.2018.01.006

22 Pan SJ, Yang Q. A Survey on Transfer Learning. *IEEE Trans Knowl Data Eng* 2010;**22**:1345–59. doi:10.1109/TKDE.2009.191

23 Vranas KC, Jopling JK, Sweeney TE, *et al.* Identifying Distinct Subgroups of ICU Patients. *Crit Care Med* 2017;**45**:1607–15. doi:10.1097/CCM.0000000000002548

24 Alaa AM, Yoon J, Hu S, *et al.* Personalized Risk Scoring for Critical Care Prognosis Using Mixtures of Gaussian Processes. *IEEE Trans Biomed Eng* 2018;**65**:207–18. doi:10.1109/TBME.2017.2698602

25 Lin J-H, Haug PJ. Exploiting missing clinical data in Bayesian network modeling for predicting medical problems. *J Biomed Inform* 2008;**41**:1–14. doi:10.1016/J.JBI.2007.06.001

26 Parker M. The Aggregation of Marginal Gains. *Bull R Coll Surg Engl* 2011;**93**:236–7. doi:10.1308/147363511X582239

27 Mador RL, Shaw NT. The impact of a Critical Care Information System (CCIS) on time spent charting and in direct patient care by staff in the ICU: A review of the literature. *Int J Med Inform* 2009;**78**:435–45. doi:10.1016/j.ijmedinf.2009.01.002

28 Bourdeaux CP, Thomas MJ, Gould TH, *et al.* Increasing compliance with low tidal volume ventilation in the ICU with two nudge-based interventions: evaluation through intervention time-series analyses. *BMJ Open* 2016;**6**:e010129. doi:10.1136/bmjopen-2015-010129

29 Ribeiro MT, Singh S, Guestrin C. 'Why Should I Trust You?': Explaining the Predictions of Any Classifier. In: *Proceedings of the 22nd ACM SIGKDD International Conference on Knowledge Discovery and Data Mining - KDD '16*. New York, New York, USA: : ACM Press 2016. 1135–44. doi:10.1145/2939672.2939778

30 Deng H. Interpreting tree ensembles with inTrees. *Int J Data Sci Anal* 2018;:1–11. doi:10.1007/s41060-018-0144-8

For peer review only

31 Holzinger A. Interactive machine learning for health informatics: when do we need the human-in-the-loop? *Brain Informatics* 2016;**3**:119–31. doi:10.1007/s40708-016-0042-6

For peer review only

Figure captions:

Figure 1: A single t-SNE embedding of the two feature matrices with each cohort plotted separately: GICU (left), and MIMIC (right). Green and red points indicate instances of RFD and NRFD respectively. The more similar two instances (in terms of feature values), the closer together they appear in the embedding space. Feature matrices displayed are those with missing entries removed.

Figure 2: Performance of the random forest (RF) and NLD classifiers over a range of prediction thresholds, for a single train:test data split. RF optimised using cross-validation with data from MIMIC and GICU (see main text). Performance evaluated on the test subset from MIMIC and GICU.

Figure 3: Performance of the four classification systems. Performance metric is the partial area under the ROC curve up to a false positive rate of 0.3. Orange lines show median pAUC value over the ensemble, while the boxes indicate the lower and upper quartiles.

For peer review only

Contributors:

CJM is the main author and conducted the data processing and analysis. RSR and DJL made important technical and methodological contributions. IDG, AC, CPB drove the study concept and design. The clinical expertise of THG, MJCT and CPB informed all stages of the project, in particular study design and interpretation of results. All authors contributed to writing the manuscript and approved the final version.

Funding:

CJM was funded by the Elizabeth Blackwell Institute Catalyst Fund. DJL is funded by Wellcome Trust and Royal Society Grant Number WT104125MA.

Acknowledgements:

We would like to thank Graeme Palmer and Amy Weaver for their support in accessing and understanding the GICU data.

Competing interests:

None declared.

Ethics approval:

University Hospitals Bristol NHS Foundation Trust audit and service evaluation.

Patient and Public Involvement :

Patients were not directly involved in this study and patient consent was not required.

Data sharing statement:

Feature matrices will be made available on Dryad. Python code for analysis and processing on request directly from the corresponding author.

For peer review only

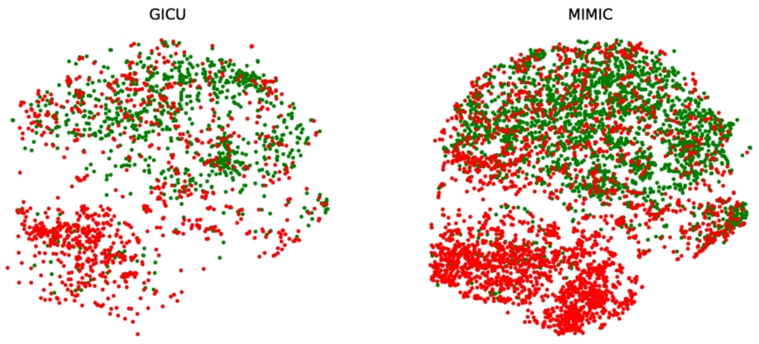

355x152mm (300 x 300 DPI)

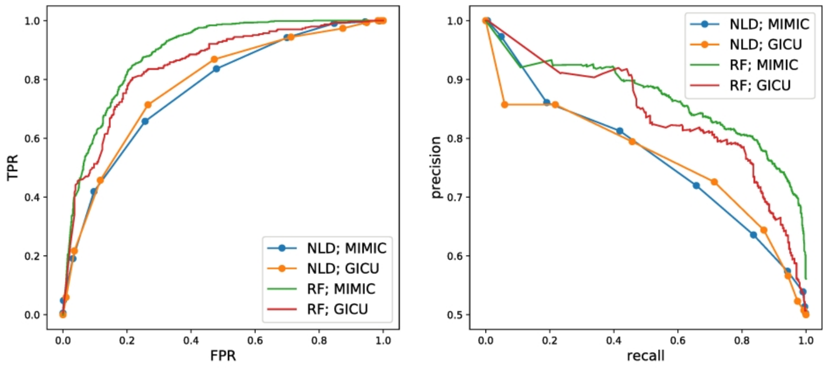

355x152mm (300 x 300 DPI)

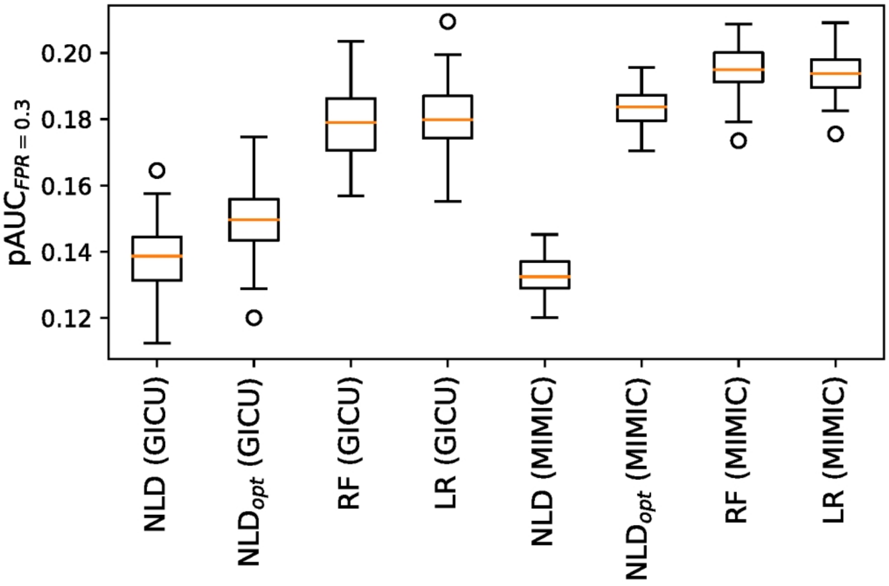

152x101mm (300 x 300 DPI)

Supplementary online file for: A machine learning approach to intensive care discharge.

Correspondance to: chris.mcwilliams@bristol.ac.uk

Contents

A Codifying discharge criteria **2**

B Data preprocessing **3**

B.1 Data harmonisation and concept mapping 3

B.2 Inclusion criteria 4

B.3 Time of day effects 6

C Feature extraction and missing data **8**

D Sensitivity of results to missing data **9**

E Implementation in code **15**

For peer review only - http://bmjopen.bmj.com/site/about/guidelines.xhtml

A Codifying discharge criteria

The original specification of the nurse-led discharge criteria [1] is presented as a check list with some room for user interpretation. For example, one of the check list questions is: *'Urea and electrolytes normal?'* In order to apply the NLD criteria to historical data we needed to codify them and remove any ambiguity. To do so we first split the original criteria into 16 binary tests, where each test places an upper and/or lower bound on a single physiological variable. If the variable value lies within the bounds the test is passed. For the *'Urea and electrolytes'* criterion we defined four separate tests: one for urea and for each of the three electrolytes identified, via clinical consultation, as most relevant to patient discharge (potassium, sodium, creatinine). We took the 'normal' values of these four variables from the bounds specified in the Philips DAR system in use on GICU.

The blood gas test (R2) was originally multi-variable, either testing the arterial blood gases (PaO2, PaCO2) or the *peripheral capillary oxygen saturation* (SpO2) in the absence of an arterial line. To simplify R2 we neglected PaO2 and PaCO2 (which were missing with high frequency), and tested only SpO2 for all patients.

The airway test (R0) checks if the patient has a patent airway. For this test we used the absence of an endotracheal tube (ETT) as a proxy for the airway being patent.

Finally, we neglected the test of urine output since this could not be successfully located in MIMIC (see section B.1).

Following this procedure we obtained the 15 codified binary tests presented in table 1 of the main text, and referred to throughout as the NLD criteria.

For peer review only - http://bmjopen.bmj.com/site/about/guidelines.xhtml

B Data preprocessing

In this section of the supplementary information we cover the details of the data preprocessing that was required prior to analysis. The basics of these procedures are outlined in the main text, and referenced to the relevant part of this section fuller for explanation.

B.1 Data harmonisation and concept mapping

Extensive pre-processing was required for both MIMIC and GICU in order to put the data in a workable format. One reason for this is that both datasets are derived from clinical information systems (CIS) that were never designed for secondary research purposes. Both MIMIC and GICU datasets contained problems with inconsistent naming conventions for physiological parameters. We determined that this problem was less severe in the portion of MIMIC-III derived from the *Metavision* data source (as compared to *Physionet*). Therefore, we restricted our study to this subset of the data. Despite this, extensive concept mapping of disparate *interventionIDs* was required in order to obtain physiological records that were coherent and as close to complete as possible. It was not possible to identify a coherent measure of urine output in MIMIC-III. This is an issue that has previously been discussed on the forums accompanying the MIMIC website. Therefore, we were forced to neglect the discharge criteria based on urine output throughout this study. In general the GICU dataset was easier to work with, in no small part thanks to working with the clinicians who are responsible for administering the associated CIS. When working with both datasets particular care was taken to ensure consistency of measurement units, which differ between the two systems.

For peer review only - http://bmjopen.bmj.com/site/about/guidelines.xhtml

B.2 Inclusion criteria

As described in the main text we selected study subjects from the MIMIC and GICU datasets according to a number of inclusion criteria. The main goal was to obtain approximately equal sized samples of both patients who were ready for discharge, and those who were not ready for discharge. The GICU data is derived from the clinical information system in use on the general intensive care unit at the Bristol Royal Infirmary. GICU is a combined critical care and high-dependency unit that treats both level 2 and level 3 patients. The MIMIC data is derived from patients admitted to critical care units at the Beth Israel Deaconess Medical Center in Boston, Massachusetts between 2001 and 2012. Initially we applied the following criteria when selecting subjects from the two historical datasets:

- From MIMIC we selected patients from MIMIC-III who were admitted to either medical or surgical intensive care, since this approximates the patient type admitted to general intensive care in Bristol.
- From MIMIC we selected only patient from *Metavision* (see section B.1).
- We only included the first intensive care stay of any given hospital admission. This decision allowed us to calculate readmission rates to ICU. (Readmission was one negative outcome considered following callout.)
- From GICU we included only patients who could be linked to Ward-Watcher (administrative system) via their lifetime ID number.
- From both data sources we selected only patients with a recorded callout (ready for discharge time).
- The two resulting cohorts of study subjects are summarised in table 2 of the main text.

For peer review only - http://bmjopen.bmj.com/site/about/guidelines.xhtml

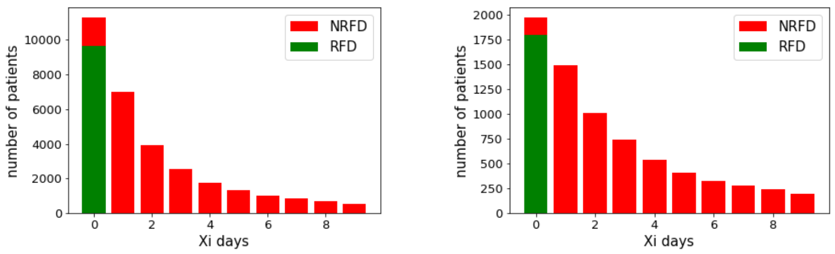

MIMIC *GICU*

Figure 1: *Illustrating instances sampled at in integer multiples (X_i) of 24 hours prior to callout. Patient callout is at $X_i = 0$. At this time patients with positive outcome are considered ready for discharge (RFD), and patients with negative outcome are considered not ready for discharge (NRFD). At $X_i > 0$ all patients are considered NRFD regardless of subsequent outcome status.*

The first step was to omit those intensive care stays with no recorded callout. This omission removed any patients who died on ICU, as well as those who should have had a callout recorded but for some reason it was missing in the data. The second step was to define positive and negative outcomes following callout (see main text). Patients with a positive outcome were determined to be ready for discharge at the time of callout. Patients with a negative outcome were determined to be not ready for discharge, either because they were erroneously discharged or because they were discharged in the knowledge that they were not fit (e.g. specialist unit transfer or palliative). For MIMIC 9627 patients had a positive outcome following callout, and were therefore deemed RFD, while 1629 patients had a negative outcome and were therefore NRFD. For GICU these numbers were 1801 and 176 patients respectively. These numbers correspond to the bars at $X_i = 0$ in figure 1.

The next step was to increase the number of instances of NRFD in order to balance to class sizes for training and testing purposes. To produce more instances of NRFD we sampled from earlier in patient stays at times when they were assumed to be not clinically fit for discharge. Heuristically we made the

For peer review only

For peer review only - http://bmjopen.bmj.com/site/about/guidelines.xhtml

decision that we should sample at least three days prior to callout to ensure no ambiguity in RFD status (one or two days prior to callout it may be the case that patients were RFD but were being held on to conservatively). From figure 1 it is clear that, given the distribution in the length of patient stays, there is only so far back in time we can sample and still obtain enough instances of NRFD to balance the class sizes. Therefore, we sampled at time points from to three to eight days prior to callout ($X_i = [3, 4, 5, 6, 7, 8]$). The reason for sampling in exact multiples of 24 hours prior to callout was to ensure the removal of time of day effects from the data (see section B.3).

Sampling all patient stays at $X_i = [3, 4, 5, 6, 7, 8]$ prior to callout yielded 8425 and 3174 instances for MIMIC and GICU respectively. We then combined these extra NRFD instances with the original instances (RFD and NRFD) sampled at time of callout ($X_i = 0$), and re-sampled the larger class to obtain a 50:50 ratio of RFD:NRFD. This re-sampling was conducted with the Python method: *SFrame.sample(fraction,seed)*. The resulting datasets for MIMIC and GICU contained 16725 and 3531 instances respectively. The subsequent removal of instances with missing data (see section C) reduced these to 5033 and 1858.

B.3 Time of day effects

Discharge decisions are invariability made in the morning (figure 2) after discussion at the morning hand over meeting and subsequent ward round. There are non-random temporal signals in the value of certain physiological variables (figure 3). Therefore, it is possible to train a reasonable classifier to identify readiness for discharge by recognising non-random variation in variable values due to time of day. For example: blood pressure is lower than average → it must be morning time → this patient is more likely to be RFD. This highlights a problem in identifying when patients are actually physically fit for discharge,

For peer review only

For peer review only - http://bmjopen.bmj.com/site/about/guidelines.xhtml

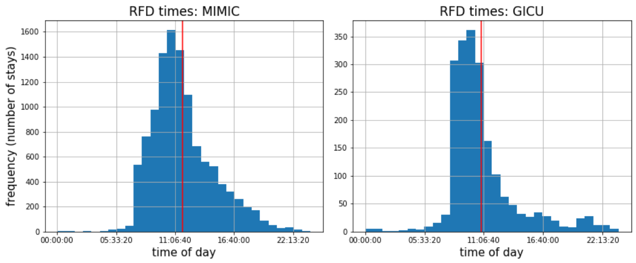

Figure 2: *Histogram of the time of day at which patients were declared ready for discharge (callout time) for: MIMIC (left) and GICU (right). The red line indicates the mean of the distribution.*

versus when they are declared so. It also means we must be careful when introducing extra instances of the negative class by sampling from earlier in patient stays - this is why we always sampled in integer multiples of 24 hours prior to callout.

The frequency with which the callout decision is taken around 11am highlights one important way that a decision support system could improve upon ICU discharge practice: by prompting clinicians to consider discharge at other, non-specified time points during the day.

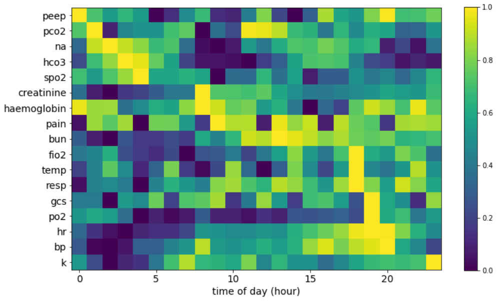

Figure 3: *Variation in average physiological variable values by time of day for the GICU dataset. We calculated the average value for a number of physiological variables at each hour of the day over all patients in the GICU dataset. The values for each variable are than scaled between 0 and 1 to be displayed on the heatmap. Certain temporal patterns become visible. For example, heart rate (HR) and blood pressure (BP) tend to be higher in the evening.*

For peer review only - http://bmjopen.bmj.com/site/about/guidelines.xhtml

C Feature extraction and missing data

We constructed feature matrices for each cohort such that the NLD criteria could easily be tested, and machine learning classifiers could be trained on the same data. In these feature matrices each row represents an instance of either RFD of NRFD, and each column represents a feature. Each of the NLD criteria tests the numeric value of a single physiological parameter. Certain criteria test whether the parameter lies within specified lower and upper bounds, while others impose only a single (lower or upper) bound (see table 1). Therefore, to evaluate a criterion with two bounds requires two feature columns: one giving the minimum value of the relevant parameter, and the other giving the maxima. A single bound criterion requires only one feature column. For example, the features 'resp_min' and 'resp_max' are required for test R4, whereas to test R1 the feature 'fio2_max' alone is sufficient.

We calculated the required minima and maxima of the physiological parameters over four-hour sample windows, as specified by the original NLD criteria. For the original instances (RFD and NRFD) this window constituted the four hours immediately preceding callout. For the extra NRFD instances this was the four hour window immediately preceding the earlier sample time (X_i days prior to callout). In both cases the resulting feature columns contained large amounts of missing data where variables had not been recorded during the specified window, as shown in table 8 in section B of the SI. This problem was worse with those variables derived from laboratory tests (such as 'bun), which are infrequently collected (approximately once every 24 hours). To replace some of the missing values the sample window was extended from four to 36 hours. If a missing variable was recorded during this extended window, the latest recorded value was taken for both the minimum and maximum. Clinically it was expected that the main source of missing data is that a variable is less likely to be recorded

For peer review only - http://bmjopen.bmj.com/site/about/guidelines.xhtml

Variables	MIMIC		GICU	
	at CALLOUT	Xi days prior to CALLOUT	at CALLOUT	Xi days prior to CALLOUT
hr_max	0.002 (0.000)	0.007 (0.005)	0.018 (0.003)	0.195 (0.194)
hr_min	0.002 (0.000)	0.007 (0.005)	0.018 (0.003)	0.195 (0.194)
spo2_max	0.011 (0.001)	0.009 (0.005)	0.027 (0.004)	0.202 (0.195)
spo2_min	0.011 (0.001)	0.009 (0.005)	0.027 (0.003)	0.202 (0.195)
airway	0.985 (0.000)	0.511 (0.000)	0.057 (0.000)	0.215 (0.000)
resp_max	0.006 (0.001)	0.009 (0.006)	0.057 (0.007)	0.203 (0.195)
resp_min	0.006 (0.001)	0.009 (0.006)	0.057 (0.007)	0.203 (0.195)
bp_min	0.007 (0.000)	0.008 (0.005)	0.069 (0.039)	0.254 (0.235)
bp_max	0.007 (0.000)	0.008 (0.005)	0.069 (0.039)	0.254 (0.235)
temp_min	0.077 (0.002)	0.079 (0.026)	0.113 (0.004)	0.229 (0.197)
temp_max	0.077 (0.002)	0.079 (0.026)	0.113 (0.004)	0.229 (0.197)
gcs_max	0.106 (0.002)	0.086 (0.011)	0.0121 (0.005)	0.233 (0.196)
gcs_min	0.106 (0.002)	0.086 (0.011)	0.0121 (0.005)	0.233 (0.196)
pain	0.461 (0.140)	0.466 (0.152)	0.194 (0.033)	0.507 (0.372)
hco3	0.885 (0.019)	0.847 (0.037)	0.490 (0.075)	0.312 (0.152)
fio2	0.932 (0.686)	0.380 (0.264)	0.767 (0.402)	0.455 (0.320)
haemoglobin	0.894 (0.024)	0.878 (0.042)	0.975 (0.016)	0.803 (0.026)
k	0.875 (0.017)	0.810 (0.034)	0.975 (0.014)	0.805 (0.028)
bun	0.884 (0.019)	0.854 (0.038)	0.975 (0.013)	0.803 (0.025)
creatinine	0.884 (0.019)	0.854 (0.037)	0.975 (0.015)	0.805 (0.027)
na	0.875 (0.017)	0.822 (0.034)	0.975 (0.013)	0.803 (0.025)

Table 1: *Summary of missing data in the MIMIC and GICU feature matrices. Values are provided for the samples drawn at time of callout, and samples drawn prior to callout (at* X_i*=[3,4,5,6,7,8]). The main values are the fraction of instances with no value recorded during the four hour window. Values in brackets give fraction of missing data when sample window is extended to 36 hours (see text).*

if it is not of clinical concern. In such cases the extended sample window represents a conservative estimation of a patient's physiological state, by taking parameter values at the last time when they were of concern to clinicians. For the five variables corresponding to double-bound NLD criteria, but with large amounts of missing data (pain, k, bun, creatinine, na) the decision was taken to produce only a single feature for each, rather than two, by taking only the final recorded value. This reduction avoided significant value duplication between feature columns.

D Sensitivity of results to missing data

In medical data there is likely to be meaning in missingness [2] i.e. data is not missing at random but often due to some other reason. For historical data it is not easy to discern the reasons for missing data, but a naive treatment of

For peer review only - http://bmjopen.bmj.com/site/about/guidelines.xhtml

missingness is liable to bias the dataset and also to lose valuable information from which to do statistical learning. In the main text we presented a complete case analysis with all patients with missing data entries removed. In this section we reproduce the results from the main text but retaining patients with fewer than three missing data entries. The missing data entries are then filled using k-nearest neighbours imputation (with $k = 5$). In this way we investigate the sensitivity of our results to missing data. Beyond this, the optimal treatment of missing data represents a significant avenue for development prior to the deployment of our methodology as a real-time decision support tool.

The inclusion of the entries with missing data does not change the following key results:

- The random forest and logistic classifiers perform better than the original NLD criteria (table 6; figures 5 and 8).
- Weighting the NLD criteria by the feature importance of the logistic classifier improves performance.
- Broadly, which features are most predictive of readiness for discharge remains unchanged from the complete case analysis (figures 6 and 7), and is consistent between classifiers (Spearman's rank correlation coefficient 0.783 here, 0.800 for complete case analysis).

The main difference introduced by the inclusion of missing data entries is a drop in performance across the board for the MIMIC cohort (table 6 and figure 8). On aggregate it becomes harder to identify MIMIC patients that are ready for discharge when those with missing entries are included. For GICU there is a slight change in the opposite direction. The NLD, random forest and logistic classifier all perform slightly better when GICU patients with missing data entries are included. These performance changes suggest non-random

For peer review only

	Predicted RFD	Predicted NRFD
RFD	11	2498
NRFD	2	2522

Table 2: *Confusion matrix for NLD criteria applied to MIMIC feature matrix, with missing data entries removed (complete case analysis).*

	Predicted RFD	Predicted NRFD
RFD	60	871
NRFD	6	921

Table 3: *Confusion matrix for NLD criteria applied to GICU feature matrix, with missing data entries removed (complete case analysis).*

	Predicted RFD	Predicted NRFD
RFD	92	8255
NRFD	13	8365

Table 4: *Confusion matrix for NLD criteria applied to MIMIC feature matrix, with missing data filled using k-nearest neighbours. Equivalent to table 2.*

	Predicted RFD	Predicted NRFD
RFD	117	1651
NRFD	10	1755

Table 5: *Confusion matrix for NLD criteria applied to GICU feature matrix, with missing data filled using k-nearest neighbours. Equivalent to table 3.*

structures in the way that data entries are missing, which likely relate to differences in clinical practice. For example it may be that MIMIC patients that are *nearly* ready for discharge a monitored less closely than more sick patients and therefore have more missing data. The inclusion of patients with missing data would introduce more of these ambiguous cases, which are nearly RFD but still being held in critical care. Although the performance changes do not alter the conclusions drawn from the results in the main text, they do suggest that strategies for dealing with missing data should be developed in accordance with on-site clinical practice when deploying such a tool in order to achieve optimal classifications.

For peer review only

For peer review only - http://bmjopen.bmj.com/site/about/guidelines.xhtml

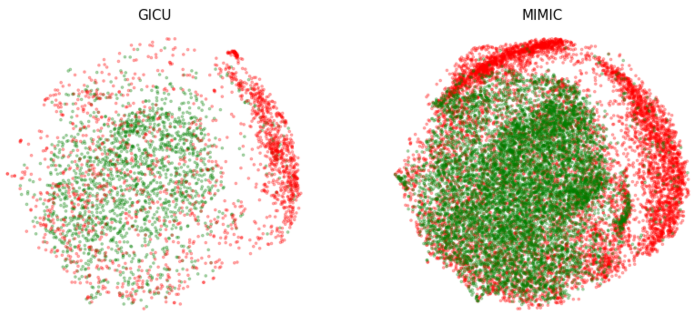

Figure 4: *t-SNE embedding [3] of the feature space for GICU (left), and MIMIC (right), with missing data filled. Green and red points indicate instances of RFD and NRFD respectively. The more similar two instances (in terms of feature values), the closer together they appear in the embedding space. Equivalent to figure 1 in the main text, although it represents a different embedding.*

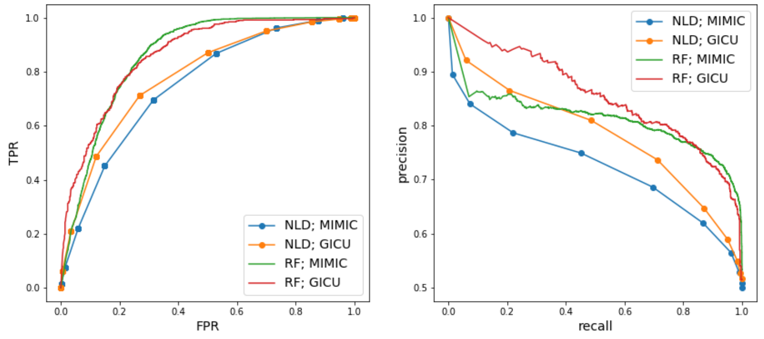

Figure 5: *Performance of the random forest (RF) and NLD classifiers over a range of prediction thresholds, for a single train:test data split. RF optimised using cross-validation with data from MIMIC and GICU (see main text). Performance evaluated on the test subset from MIMIC and GICU. Left: ROC curves. Right: PRC curves. Equivalent to figure 2 in the main text.*

For peer review only - http://bmjopen.bmj.com/site/about/guidelines.xhtml

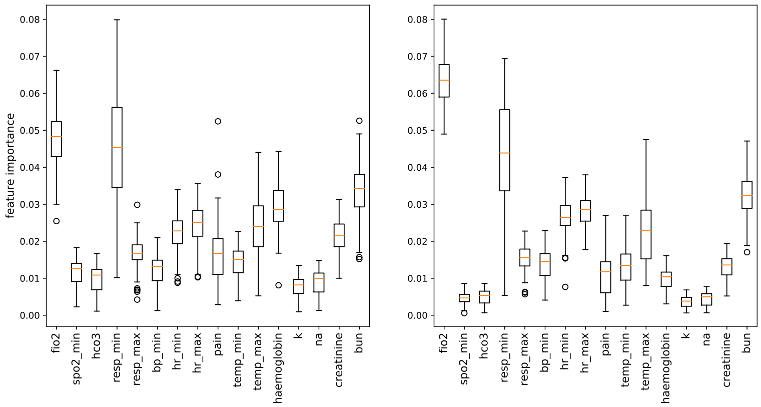

Figure 6: *Comparison between the feature importances of the random forest classifier on trained on two different datasets. Left: all entries with missing data removed (as for results presented in main text). Right: entries with two or less missing data values retained, missing values filled using k-nearest neighbours imputation (see text in appendix D). Feature importances calculated over ensemble of 100 train:test data splits. Note: the dominant features 'airway' and 'gcs_min' are not shown, for visual clarity of the other features.*

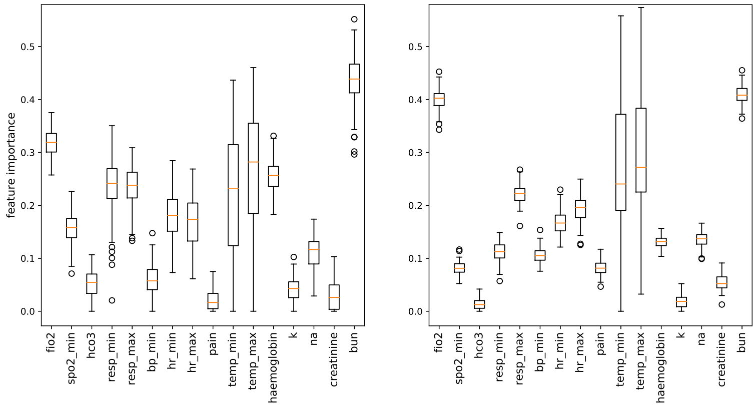

Figure 7: *Equivalent to figure 6 but for the logistic classifier. Note: the dominant features 'airway' and 'gcs_min' are not shown, for visual clarity of the other features.*

For peer review only - http://bmjopen.bmj.com/site/about/guidelines.xhtml

Test data	NLD	$NLD_{weighted}$	Random forest	Logistic classifier
MIMIC	0.444(±0.017)	0.610(±0.020)	0.651(±0.022)	0.646(±0.021)
GICU	0.460(±0.031)	0.502(±0.035)	0.595(±0.035)	0.601(±0.034)
MIMIC (knn-fill)	0.410(±0.010)	0.490(±0.013)	0.565(±0.015)	0.540(±0.014)
GICU (knn-fill)	0.483(±0.023)	0.523(±0.026)	0.603(±0.022)	0.622(±0.024)

Table 6: *Performance summary of the four classification systems: the original nurse-led discharge criteria (NLD), a weighted version of the criteria ($NLD_{weighted}$), the random forest, and the logistic classifier. $NLD_{weighted}$ is obtained by weighting the NLD criteria with feature importance values obtained from the logistic classifier. Metric reported is the normalised partial area under the ROC curve (pAUC), up to a false positive rate of 0.3 (see main text). Performance averaged over 100 different train:test data splits. Mean(± standard deviation) is reported.*

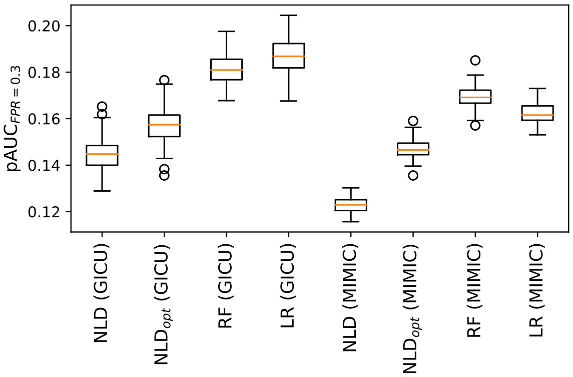

Figure 8: *Equivalent to figure 3 in main text, but for data with missing entries retained (knn-fill).*

For peer review only - http://bmjopen.bmj.com/site/about/guidelines.xhtml

E Implementation in code

Data extraction and preprocessing were conducted using SQL. We worked with a local MySQL instance of the MIMIC-III data, and the Bristol (GICU) data was accessed from the backend of the Philips DAR clinical information system via Microsoft SQL Reporting Services on the hospital server. Additional patient demographic information was obtained from the WardWatcher system, which is the ICNARC reporting software in use on the general intensive care unit in Bristol. Entries from WardWatcher were linked to the Philips data via patient lifetime identification numbers. Subsequent data processing and analysis was conducted in Python. We used the machine learning package scikit-learn to train and test classifiers. In particular the method *GridsearchCV* was used to optimise model hyperparamaters via cross-validation, optimising for the F1-score. This optimisation was conducted on a predefined data split, using only MIMIC data for training and only GICU data for validation. The 'best_estimator' parameter of *GridsearchCV* was set to 'True' such that the optimised model was refitted to the full training data (MIMIC and GICU). For the logistic classifier the regularisation hyperparameter 'c' was optimised over a logarithmic range of twenty values between 1×10^{-3} and 1×10^{3}. For the random forest three hyperparameters were optimised over the following ranges: $n\_estimators \in [20, 50, 100]$; $max\_features \in [20, 50, 100]$; $max\_depth \in [4, 5, 6, 7]$.

For peer review only - http://bmjopen.bmj.com/site/about/guidelines.xhtml

References

[1] Gillian Knight. Nurse-led discharge from high dependency unit. *Nursing in critical care*, 8(2):56–61, 2003.

[2] Jau-Huei Lin and Peter J Haug. Exploiting missing clinical data in bayesian network modeling for predicting medical problems. *Journal of biomedical informatics*, 41(1):1–14, 2008.

[3] Laurens van der Maaten and Geoffrey Hinton. Visualizing data using t-sne. *Journal of Machine Learning Research*, 9(Nov):2579–2605, 2008.

For peer review only

For peer review only - http://bmjopen.bmj.com/site/about/guidelines.xhtml

BMJ Open

Towards a decision support tool for intensive care discharge: machine learning algorithm development using electronic health care data from MIMIC-III and Bristol, UK.

Journal:	*BMJ Open*
Manuscript ID	bmjopen-2018-025925.R1
Article Type:	Research
Date Submitted by the Author:	20-Nov-2018
Complete List of Authors:	McWilliams, Christopher; University of Bristol, Engineering Mathematics<br>Lawson, Daniel; University of Bristol, Integrative Epidemiology Unit, Population Health Sciences<br>Santos-Rodriguez, Raul; University of Bristol, Engineering Mathematics<br>Gilchrist, Iain; University of Bristol, Department of Experimental Psychology<br>Champneys, Alan; University of Bristol, Engineering Mathematics<br>Gould, Timothy; University Hospitals Bristol NHS Foundation Trust, Intensive Care Unit<br>Thomas, Mathew; University Hospitals Bristol NHS Foundation Trust, Intensive Care Unit<br>Bourdeaux, Christopher; University Hospitals Bristol NHS Foundation Trust, Intensive Care Unit
**Primary Subject Heading**:	Intensive care
Secondary Subject Heading:	Health informatics
Keywords:	Adult intensive & critical care < ANAESTHETICS, Clinical audit < HEALTH SERVICES ADMINISTRATION & MANAGEMENT, Health informatics < BIOTECHNOLOGY & BIOINFORMATICS, Information technology < BIOTECHNOLOGY & BIOINFORMATICS

SCHOLARONE™
Manuscripts

Towards a decision support tool for intensive care discharge: machine learning algorithm development using electronic health care data from MIMIC-III and Bristol, UK.

Christopher J. McWilliams*[1], Daniel J. Lawson[2], Raul Santos-Rodriguez[1], Iain D Gilchrist, Alan Champneys[1], Timothy H. Gould[4], Matthew J.C. Thomas[4], and Christopher P. Bourdeaux[4]

1 Department of Engineering Mathematics, University of Bristol
2 Integrative Epidemiology Unit, Population Health Sciences, University of Bristol
3 School of Experimental Psychology, University of Bristol
4 Intensive Care Unit, Queens Building, University Hospitals Bristol

*Corresponding author: chris.mcwilliams@bristol.ac.uk

Keywords: patient discharge, machine learning, clinical decision support, critical care, patient flow

Word count: 3746 (main text only)

For peer review only

Abstract

Objective
The primary objective is to develop an automated method for detecting patients that are ready for discharge from intensive care.

Design
We used two datasets of routinely collected patient data to test and improve upon a set of previously proposed discharge criteria.

Setting
Bristol Royal Infirmary general intensive care unit (GICU).

Patients
Two cohorts derived from historical datasets: 1870 intensive care patients from GICU in Bristol, and 7592 from MIMIC-III (a publicly available intensive care dataset).

Results
In both cohorts few successfully discharged patients met the of all the discharge criteria. Both a random forest and a logistic classifier, trained using multiple-source cross-validation, demonstrated improved performance over the original criteria and generalised well between the cohorts. The classifiers showed good agreement on which features were most predictive of readiness-for-discharge, and these were generally consistent with clinical experience. By weighting the discharge criteria according to feature importance from the logistic model we showed improved performance over the original criteria, while retaining good interpretability.

Conclusions
Our findings indicate the feasibility of the proposed approach to ready-for-discharge classification, which could complement other risk models of specific adverse outcomes in a future decision support system. Avenues for improvement to produce a clinically useful tool are identified.

Strengths and Limitations of this study:

- Machine learning techniques demonstrate improved performance over a set of clinical criteria in classifying patients that are ready for discharge.
- Training data from multiple source domains is leveraged to produce general classifiers.
- The restrictive feature representation tested could be expanded to better exploit the richness of available data and boost performance.
- Our approach has the potential to streamline the discharge process in cases where patient physiology makes them clear candidates for a de-escalation of care.
- High-risk patients would require additional levels of decision support to facilitate complex discharge planning.

For peer review only

Introduction

Demand for intensive care unit (ICU) beds is rising at a time when the resource is constrained[1]. In order to optimise the allocation of this resource, patients should be discharged from the ICU as soon as they no longer require the specialist input provided there. The reduced ICU capacity caused by discharge delay can result in the delayed admission of patients requiring critical care[2,3]. Furthermore, patients remaining in the ICU after they are medically fit to leave are at risk of iatrogenic harm and may experience detrimental effects on physical rehabilitation and psychosocial well-being[4].

The identification of individuals that are ready to leave ICU is a key component of patient flow through the hospital. At present this identification is a manual process, relying on physicians reviewing patients on a ward round at a standard point in time. There is a lack of formal guidance to inform discharge readiness and as such the process is sensitive to both the decision making heuristics of individual clinicians and structural factors within the hospital[5]. A number of studies have looked to address this problem by attempting to standardise the discharge process. In a scoping review of these studies Stelfox et al.[6] noted that, while a range of tools have been developed to characterise discharge readiness, most studies have been single-centre and have not conducted comparative evaluations of different tools.

Increasingly ICUs are using clinical information systems (CIS) to collect, store and display physiological data. The availability of such routinely collected patient data presents the opportunity to apply methods from data science, with the potential to transform healthcare in a number of ways[7,8]. Two particular avenues for development are the automation of simple tasks[9] and the implementation of decision support systems[10], both of which could reduce the cognitive load of clinicians and free up scarce resource for tasks that require human expertise. This work considers the ICU discharge process, which has accessible data from routine collection and requires a simple but important binary decision that could benefit from an evidence-based approach. Indeed, several statistical models have recently been developed to predict the risk of adverse events following intensive care discharge[11–15]. Such risk models are invaluable tools for clinical decision making and, in the context of ICU discharge, can provide information with which to plan complex de-escalations of care. For example, patients deemed to be at high-risk of readmission may benefit from continued close monitoring[16], since early detection of deterioration is a strong predictor of outcome[17,18].

In our previous work on the psychology of clinical decision making we have demonstrated the effectiveness of simple 'nudge' based interventions in changing clinical practice[19–21]. Building on this foundation we were motivated to develop a classifier to automatically flag patients that appear physiologically fit for discharge. The intention is that such a screening tool could streamline morning ward rounds by allowing staff to focus their attention on the most likely-dischargeable patients. The tool could also prompt clinicians to consider discharge decisions at other times of day, outside of normal rounds. In 2003 Knight proposed a set of nurse-led discharge criteria[22] with a similar aim – to expedite discharge from a high-dependency unit by allowing nurses to discharge patients who were clearly well enough to leave. These criteria represent a general and highly conservative set of constraints on physiology that characterise a patient as suitable for care on an acute ward (level 1 care). High-risk patients are unlikely to meet these criteria, but may still be dischargeable by a consultant. In this study we use routinely collected patient data to retrospectively evaluate Knight's criteria, and then improve upon their performance using machine learning methods. To this end we study two historical cohorts. One cohort consists of patients treated on the general intensive care unit at the Bristol Royal Infirmary between January 2015 and April 2017,

For peer review only

while the second consists of patients selected from the MIMIC-III database[23] (see Methods for details).

For peer review only

Methods

Discharge criteria

The nurse-led discharge (NLD) criteria proposed by Knight[22] consist of a set of constraints on various routinely collected vital signs and laboratory results. If a patient meets all the criteria for a period of at least four hours, Knight states that they may be safely discharged by a nurse. The motivation behind developing these criteria was to facilitate discharge by nurses in cases where the decision was clear, and there is some evidence of improved bed allocation when using such a nurse-led system[22,24,25]. In order to test the NLD criteria on historical patient data we codified the constraints (see online supplementary file section A) into 15 binary tests, which are defined in table 1. For criteria that were not assigned numeric values in the original publication (B1-4, CNS) we used the 'normal' bounds as defined in our clinical information system.

Table 1: Codified version of the discharge criteria for application to electronic health record data. Here the fifteen criteria have been grouped into intuitive subsets and each assigned a test ID ('R0' to 'B4'). According to the original specification, if all 15 criteria are met for a period of at least four hours the patient can be safely discharged.

Test ID	Test name	Variable	Test condition
**R0**	Respiratory: airway	airway	airway patent
**R1**	Respiratory: FiO2	fio2	fio2 ≤ 0.6
**R2**	Respiratory: blood oxygen	spo2	spo2 ≥ 95 (%)
**R3**	Respiratory: bicarbonate	hco3	hco3 ≥ 19 (mmol/L)
**R4**	Respiratory: rate	resp (rate)	10 ≤ resp ≤ 30 (bpm)
**C0**	Cardiovascular: blood pressure	bp (systolic)	bp ≥ 100 (mmHg)
**C1**	Cardiovascular: heart rate	hr	60 ≤ hr ≤ 100 (bpm)
**P**	Pain	pain	0 ≤ pain ≤ 1
**CNS**	Central nervous system	gcs	gcs ≥ 14
**T**	Temperature	temp	36 ≤ temp ≤ 37.5 (C)
**B0**	Bloods: haemoglobin	haemoglobin	haemoglobin ≥ 9 (g/dL)
**B1**	Bloods: potassium	k	3.5 ≤ k ≤ 6.0 (mmol/L)
**B2**	Bloods: sodium	na	130 ≤ na ≤ 150 (mmol/L)
**B3**	Bloods: creatinine	creatinine	59 ≤ creatinine ≤ 104 (umol/L)
**B4**	Bloods: urea	bun	2.5 ≤ bun ≤ 7.8 (mmol/L)

Cohort selection

Subjects for this study were selected from two distinct historical data sources to form two patient cohorts. The inclusion criteria are detailed in section B of the online supplementary file. The first data source consists of the routinely collected data from 1870 patients treated on the general intensive care unit at the Bristol Royal Infirmary. We refer to the cohort selected from this dataset as *GICU*. The second data source was derived from the MIMIC-III database[23], from which we selected patients who were admitted to medical or surgical intensive care since this approximates the patient type in GICU. We restricted our analysis to the 'Metavision' subset of MIMIC-III, since the labelling of the variables required to evaluate the NLD criteria was found to be more consistent in this portion of the database. Furthermore, we selected only the first intensive care stay of any given hospital admission, and only those stays for which there was a recorded *callout* (ready-for-

discharge) time. Following these criteria, we arrived at a subset of 7592 patients from MIMIC-III, forming the cohort we refer to hereafter as *MIMIC*.

The use of two cohorts was motivated by two concerns. Firstly, by including the MIMIC cohort, we significantly increased the volume of data available for training classifier algorithms. Secondly, the use of two cohorts allowed us to study the generalisation of our results between different patient populations under different healthcare systems.

Readiness-for-discharge

The key to testing and improving on the discharge criteria was to be able to identify, from the historical data, patients that were ready-for-discharge (RFD) and not-ready-for-discharge (NRFD). Whereas previous models have looked to predict the occurrence of adverse events following ICU discharge[12,15] we wanted to learn to classify those patients that appear physiologically fit to leave the unit. These are subtly different tasks. The former requires the identification of patients at risk of negative outcomes from those who have already been declared fit for discharge, while the later looks to identify, from a sample of ICU patients, those who are no longer in need of critical care. Clearly the latter is an easier task. In order to train a classifier for this task it was necessary to define instances of the positive (RFD) and negative classes (NRFD). Both datasets (GICU and MIMIC) contain a callout for each patient, which marks the time at which a patient was declared clinically ready to leave the intensive care unit. A patient was defined as RFD at their time of callout, provided they had a positive outcome after leaving ICU. Conversely, patients with a negative outcome were defined as NRFD at their time of callout. A positive outcome was defined as the patient leaving hospital alive without readmission to ICU. A negative outcome was defined as either readmission to ICU during the same hospital admission, or in-hospital mortality after discharge from ICU. We note that it is more conventional to use readmission (or mortality) within 48 hours to define a negative outcome related to ICU care[12,26]. However, this practice is not universal[27] and in our case it was not possible because of limitations in the data available locally.

Table 2: Patient characteristics for the two cohorts. Discharge delay defined as length of time between callout and discharge from ICU. Readmission to ICU defined as readmission during same hospital stay. Negative outcome is in-hospital mortality and/or readmission.

	MIMIC	GICU
**Total patients**	7592	1870
**Gender, % female**	47.6	40.5
**Age, median years (IQR))**	64.0 (50.9-77.0)	63.0 (49.0-72.8)
**BMI, median (IQR)**	28.1 (24.9-31.6)	26.5 (22.8-30.6)
**Length of stay, median days (IQR)**	1.93 (1.11-3.34)	2.96 (1.69-5.14)
**Discharge delay, median days (IQR)**	0.27 (0.18-0.39)	0.34 (0.20-1.04)
**In-hospital mortality, # (%)**	466 (6.14)	67 (3.58)
**Readmission to ICU, # (%)**	589 (7.76)	52 (2.78)
**Negative outcomes, # (%)**	954 (12.57)	109 (5.83)

For peer review only

Given the low rates of negative outcome following callout in both MIMIC and GICU (see table 2), we generated further instances of the negative class, in order to balance the class sizes. Conceptually this is equivalent to providing more instances for the classifier to learn the physiological characteristics of patients requiring ongoing critical care. To do this we sampled patients at between three and eight days prior to their callout (see supplementary section B: figures 1-3), under the assumption that patients were not-ready-for-discharge at this point in time, regardless of their eventual outcome state (positive or negative). Patients within the first 24 hours of their ICU stay were omitted from this sample. Full details of the sampling procedure are given in section B of the online supplementary file.

Feature extraction

We used the same feature set to evaluate the NLD criteria and to train machine learning classifiers.We constructed either one or two features corresponding to each of the NLD criteria, depending on the criteria in question and on data availability. For example, the features 'resp min' and 'resp max' were used to test the criterion R4, whereas the single feature 'bun' was used to test B4. Where possible the feature values were calculated from a four hour sample window, as specified by the original NLD criteria. In the cases where no data was available during the four hour window, an extended 36 hour window was used. This extended window was mainly relevant for infrequently measured laboratory test results (see table 1 in section C of the online supplementary file). Full details and justification of the feature extraction procedure are provided in section C of the online supplementary file. Since this feature set is somewhat restrictive, consisting of 18 physiological features, we also defined an *extended* feature set that included the following extra features: *age, sex, BMI* and *hours since admission*.

To produce the results presented in the main text, missing feature values were imputed using k-nearest neighbour imputation[28]. Full details of the imputation procedure are given in section D of the online supplementary file, along with a complete case analysis that addresses the sensitivity of our results to this imputation strategy. When training and testing the machine learning classifiers, features were standardised by subtracting the mean and dividing by the standard deviation. The feature matrices for the imputed and complete case data sets are visualised using the t-SNE algorithm[29] in figures 4 and 5 of section D in the online supplementary file.

Analysis of NLD criteria

Knight originally specified that all 15 criteria must be met in order to allow safe discharge by a nurse[22]. Following this specification we evaluated the criteria for both MIMIC and GICU, determining which instances were classified as RFD and NRFD, and comparing these results to ground-truth. We then further investigated the performance of the NLD criteria as a classification system, by relaxing the constraint that all 15 tests must be passed in order to make an RFD classification. Instead we used the NLD criteria to produce probability estimates of being RFD, by summing the number of tests passed and dividing by 15 to produce a normalised output between 0 and 1. In this formulation each of the 15 criteria contribute equally to the RFD probability. Subsequently we weighted each of the criteria according to a measure of feature importance (see below) in order to improve their predictive performance. Using the probability outputs it was possible to evaluate the performance of the NLD criteria in the same way as the machine learning classifiers described below.

Machine learning classifiers

For peer review only

To improve upon the performance of the NLD criteria, we trained and tested two machine learning classifiers: a random forest (RF)[30], and a logistic classifier (LC)[31]. These two algorithms were chosen for their simplicity in implementation and ease of interpretation in their predictive output. The training methodology we used was intended to produce classifiers that made good use of the training data that comes from multiple source domains, whilst generalising well to new patient populations. As such we employed multiple-source cross-validation[32]. A single iteration of this procedure is as follows. Each source dataset is split into train and test data. For GICU 30% of the data is held out for testing. For MIMIC an equal sized test set is held out (~10%). Multiple-source cross-validation is then used to optimise the hyper-parameters on the training data (see section E of the online supplementary file) with two folds, one derived entirely from MIMIC and the other derived entirely from GICU. The optimised classifier is then retrained on the full training data (MIMIC and GICU), and its performance is tested on the held-out test data. This procedure is repeated over 100 random train-test splits to produce estimates of the mean and standard deviation of classifier performances.

In order to determine the feature importances for each classifier, and therefore understand which features were most predictive of readiness-for-discharge, we calculated the permutation feature importance[33]. In short this procedure involves iterative random permutation of the values of each feature, and the calculation of average loss of classifier performance (we used area under the ROC curve) resulting from this feature randomisation. The overall performance of our trained classifiers, and the NLD criteria, was characterised by producing receiver-operator-characteristic (ROC) and precision-recall (PRC) curves[34], and by evaluating a suite of common performance metrics.

For peer review only

Results.

The original specification of the NLD criteria proved to be highly conservative as expected, producing low false positive and true positive rates for both cohorts (supplementary section D: tables 2-5). The true positive rates for MIMIC and GICU were 1.1% and 6.6% respectively. Varying the threshold number of criteria required to make an RFD classification allowed us to produce ROC and PRC curves for the NLD criteria. These curves are illustrated in figure 1 for a single train-test data split. On this data split the NLD criteria obtained precisions of greater than 0.7 up to a recall of 0.6 for both cohorts. The random forest using the extended feature set showed large performance gains on this data split, with precisions of greater than 0.8 up to a recall of 0.8.

Table 3: Performance metrics for the various classification systems. All scores are averaged over 100 train-test data splits and given as: mean (standard deviation). All metrics other than AUROC and Brier score are evaluated at a specificity of 0.7, using linear interpolation to estimate this operating point in ROC-space. $NLD_{\_weighted}$ are the NLD criteria, weighted by feature importances from the logistic classifier. $LC_{\_extended}$ and $RF_{\_extended}$ are the machine learning classifiers with extended feature sets.

		NLD	$NLD_{\_weighted}$	LC	RF	$LC_{\_extended}$	$RF_{\_extended}$
GICU	AUROC	0.7913 (0.0098)	0.8197 (0.0098)	0.8788 (0.0087)	0.8692 (0.0093)	**0.8822 (0.0091)**	0.8721 (0.0094)
	Accuracy	0.7222 (0.0248)	0.7829 (0.0339)	0.8397 (0.0492)	0.8389 (0.0496)	0.8318 (0.0475)	**0.8426 (0.0505)**
	F1	0.7473 (0.0109)	0.7709 (0.0153)	0.8109 (0.0099)	0.8102 (0.0115)	0.8050 (0.0119)	**0.8129 (0.0109)**
	Specificity	0.7000 (0.0000)	0.7000 (0.0000)	0.7000 (0.0000)	0.7000 (0.0000)	0.7000 (0.0000)	0.7000 (0.0000)
	pAUROC	0.1469 (0.0061)	0.1471 (0.0076)	0.1961 (0.0068)	0.1876 (0.0078)	**0.1989 (0.0068)**	0.1888 (0.0079)
	Brier	0.2677 (0.0060)	0.2265 (0.0083)	0.1465 (0.0052)	0.1502 (0.0056)	**0.1439 (0.0059)**	0.1482 (0.0049)
	Sensitivity	0.7426 (0.0166)	0.8098 (0.0263)	0.8870 (0.0171)	0.8860 (0.0196)	0.8767 (0.0196)	**0.8909 (0.0185)**
MIMIC	AUROC	0.7442 (0.0059)	0.8248 (0.0056)	0.8549 (0.0124)	0.8605 (0.0122)	0.8726 (0.0108)	**0.8859 (0.0110)**
	Accuracy	0.6783 (0.0125)	0.8007 (0.0358)	0.8366 (0.0513)	0.8387 (0.0517)	0.8494 (0.0533)	**0.8531 (0.0545)**
	F1	0.6908 (0.0120)	0.7830 (0.0103)	0.8084 (0.0171)	0.8097 (0.0158)	0.8175 (0.0123)	**0.8201 (0.0133)**
	Specificity	0.7000 (0.0000)	0.7000 (0.0000)	0.7000 (0.0000)	0.7000 (0.0000)	0.7000 (0.0000)	0.7000 (0.0000)
	pAUROC	0.1238 (0.0030)	0.1429 (0.0043)	0.1677 (0.0100)	0.1729 (0.0099)	0.1837 (0.0092)	**0.1955 (0.0091)**
	Brier	0.2510 (0.0029)	0.1986 (0.0046)	0.1470 (0.0065)	0.1472 (0.0069)	0.1394 (0.0056)	**0.1388 (0.0064)**
	Sensitivity	0.6713 (0.0126)	0.8337 (0.0174)	0.8827 (0.0282)	0.8860 (0.0265)	0.9001 (0.0207)	**0.9049 (0.0210)**

In general the machine learning classifiers outperformed the NLD criteria. These performances, averaged over all 100 train-test data splits are summarised in table 3. The random forest performed better than the logistic classifier on MIMIC, according to all performance metrics, when using both the original and extended feature sets. On GICU the random forest and logistic classifier produced similar scores. For this cohort, the logistic classifier with the original feature set narrowly outperformed the random forest according to all metrics, but only won on three metrics (AUROC, pAUROC and Brier score) when the extended feature set was used. Overall, when training and testing on the imputed dataset, the random forest with extended feature set showed the best performance. The complete case analysis (table 6 in section D of the online supplementary file) produced qualitatively similar results but there was a clearer distinction between classifiers, with the logistic classifier performing better on GICU and the random forest performing better on MIMIC.

For peer review only

Average receiver operating characteristics are summarised for all classifiers in tables 7 and 8 of section D in the online supplementary file.

Table 4: Feature importances given by the random forest (RF) and logistic classifier (LC), evaluated over 100 train-test data splits. Importance values are given as: mean (standard deviation). Features are ranked according to mean importance value, and the table is ordered according to the ranking given by the logistic classifier.

	Importance (LC)	Importance (RF)	Rank (LC)	Rank (RF)
**gcs_min**	0.1053 (0.0026)	0.1029 (0.0102)	0	0
**airway**	0.0776 (0.0026)	0.0602 (0.0076)	1	1
**bun**	0.0190 (0.0009)	0.0139 (0.0025)	2	3
**fio2**	0.0096 (0.0006)	0.0205 (0.0024)	3	2
**hr_max**	0.0063 (0.0009)	0.0076 (0.0015)	4	4
**haemoglobin**	0.0061 (0.0006)	0.0040 (0.0014)	5	6
**resp_max**	0.0037 (0.0006)	0.0031 (0.0010)	6	7
**hr_min**	0.0024 (0.0006)	0.0047 (0.0014)	7	5
**na**	0.0010 (0.0003)	0.0005 (0.0004)	8	15
**hco3**	0.0009 (0.0003)	0.0006 (0.0005)	9	14
**spo2_min**	0.0005 (0.0002)	0.0005 (0.0003)	10	16
**bp_min**	0.0003 (0.0001)	0.0013 (0.0009)	11	11
**resp_min**	0.0001 (0.0001)	0.0020 (0.0007)	12	9
**pain**	0.0000 (0.0000)	0.0009 (0.0006)	13	13
**creatinine**	0.0000 (0.0000)	0.0028 (0.0011)	14	8
**k**	0.0000 (0.0000)	0.0003 (0.0003)	15	17
**temp_min**	0.0000 (0.0000)	0.0012 (0.0009)	16	12
**temp_max**	0.0000 (0.0000)	0.0018 (0.0008)	17	10

Broadly the two classifiers agreed as to which features were important in classifying patients as ready-for-discharge (table 4). Eight of the features ranked in the top ten by the logistic classifier were also ranked in the top ten by the random forest, and the Spearman's rank correlation coefficient between the feature rankings was 0.761 (p=0.00002). Both classifiers ranked *gcs_min* and *airway* as the two most important features by a significant margin. There was little change in these feature rankings under the complete case analysis (table 9 in section D of the online supplementary file). We attempted to improve the classification performance of the NLD criteria by weighting each of the criteria according to the corresponding feature importance given by the logistic classifier. This weighting produced small performance gains over the original criteria (see $NLD_{weighted}$ in table 3), but not enough to warrant their use instead of a machine learning classifier in a clinical setting.

For peer review only

Discussion

Identifying which patients are suitable for ICU discharge is complex[1,6]. Delayed and out of hours discharges are associated with increased mortality[35], and patients in ICU who could be managed on the ward put an unnecessary strain on resources. The determination of ready-for-discharge status is influenced by many unmeasured factors, such as ICU census[25],and this leads to unwarranted variation in clinical decision making. Furthermore, the decision to declare someone fit for discharge is based on the judgement of individual clinicians and is likely to be given a lower priority than decisions about treatment options for patients who are more unwell.

In this study we have put forwards the concept of a decision support tool that would prompt clinicians to consider discharging a patient when they appear physiologically ready-for-discharge. Such a prompt would occur by means of a dashboard notification, or 'nudge'[20]. It would need to be sufficiently sensitive as to recommend high numbers of potential discharges, whilst providing enough specificity to retain clinician engagement. Here we have detailed the development of two machine learning algorithms intended for such a purpose, and demonstrated their performance improvement over a previously published set of criteria that were originally aimed at discharge automation[22]. At a threshold specificity of 0.7, the algorithm with best overall performance achieved mean sensitivities of 0.8909 and 0.9049 for the GICU and MIMIC cohorts respectively (table 7 in section D of the online supplementary file). This represents a relatively high rate of false positives and suggests that further development is required before a tool based on this approach could be deployed clinically.

The features identified as most important by the classifiers were clinically meaningful. Clinicians will recognise that coma score, respiratory function and renal function are strongly related to successful ICU discharge. Under the logistic classifier certain features, such as body temperature and creatinine, appeared to be less important than we expected. This may be, in part, a consequence of patient heterogeneity on the general intensive care unit[36]. For example, body temperature may be predictive for patients with infection yet much of this predictive power is lost in our attempt to fit a general model for the whole ICU population. Similarly, although creatinine levels are indicative of renal function, persistently high creatinine should not be a criterion against discharge readiness for patients with chronic renal failure. The ability of the random forest to better model such non-linear feature dependencies may explain why it gave a higher rank to these features.

In general, the performance of both classifiers would benefit from expanding the feature representation. The feature set we used was chosen to be directly analogous with the features tested by the discharge criteria. This feature set is restrictive, having been originally designed to be manually recorded by nurses using paper charts. We demonstrated that adding four extra features (age, sex, BMI and hours since admission) improved classification performance. However, machine learning methods have the power to further exploit the richness of the data held in electronic charting systems by including more physiological parameters, and learning the most predictive feature representation of these parameters (see for example [37]). One barrier to this approach is the challenge of harmonising the data, especially when combining data from different sources. This is one reason that we did not include diagnosis codes or severity of illness scores in this study, although they have previously been shown to be predictive of adverse events following discharge[11,12]. During a patient's stay in ICU, many of their physiological parameters are controlled by clinical intervention, and their expected physiological state is dependent on their medical history (see, for example, guidelines on acceptable levels of Hb in different patient types[38]). Therefore, conditioning features on medical interventions and applying methods for patient sub-typing[36,39] are two improvements that we expect could significantly boost performance. Also, although the complete case analysis did not qualitatively alter our results, the

development of a more sophisticated multiple-imputation strategy[40] would likely improve performance by making best use of the available training data and exploiting the value in missingness[41].

A range of different tools and methods have previously been proposed with the aim of improving ICU discharge practice. These tools range from criteria to evaluate discharge readiness[22,42], to guidelines for discharge planning and education[6]. Additionally, a number of risk models have been developed to predict adverse outcomes following ICU discharge[11–13,15,43]. In particular Badawi and Breslow demonstrated that mortality and readmission should be modelled independently as separate outcomes[12]. Clearly a comparative evaluation of the existing tools is required in a clinical setting. We argue that a future decision support system for discharge planning should draw elements from all these methods. A screening algorithm, such as the one we have outlined here, could notify clinicians of dischargeable patients in cases where the decision is clear. Decisions around high-risk patients, which are frequently required, would benefit from an extra level of decision support, such as individual predictions of mortality and readmission risk[12]. The increasing availability of intensive care research datasets[44,45] is sure to improve the performance and generality of such models, particularly as methods from transfer learning are applied[15]. Ultimately the benefit from these models comes from the manner in which they are deployed. We have shown in previous work that subtle changes to the presentation of information can have significant impact on clinical decision making[20]. The aggregate effects of the small improvements produced by such approaches could be widely beneficial[46]. We suggest that the proposed decision support system would maximise engagement by addressing issues of model interpretability[47,48], and could leverage clinical expertise by learning online with a human-in-the-loop[49].

For peer review only

Conclusion

This work outlines a framework for the use of machine learning algorithms to identify patients that are physiologically fit for discharge from the ICU. A decision support tool based on these methods could contribute to the solution of this significant clinical and operational problem by streamlining the discharge process and reducing unnecessary ICU stay. We have identified a number of improvements that would be required before the deployment and testing of such a tool in a clinical setting, and highlighted how the tool would benefit from the inclusion of multiple complementary modelling frameworks. As more patient data becomes available in the wider hospital setting there is extensive scope to use data-driven methods, such as the one presented here, to improve patient flow through hospitals.

For peer review only

References:

1 Rubenfeld GD, Rhodes A. How many intensive care beds are enough? *Intensive Care Med* 2014;**40**:451–2. doi:10.1007/s00134-014-3215-x

2 Chalfin DB, Trzeciak S, Likourezos A, *et al.* Impact of delayed transfer of critically ill patients from the emergency department to the intensive care unit. *Crit Care Med* Published Online First: 2007. doi:10.1097/01.CCM.0000266585.74905.5A

3 Cardoso LT, Grion CM, Matsuo T, *et al.* Impact of delayed admission to intensive care units on mortality of critically ill patients: a cohort study. *Crit Care* 2011;**15**:R28. doi:10.1186/cc9975

4 Howell MD. Managing ICU throughput and understanding ICU census. *Curr Opin Crit Care* 2011;**17**:626–33. doi:10.1097/MCC.0b013e32834b3e6e

5 Capuzzo M, Moreno RP, Alvisi R. Admission and discharge of critically ill patients. *Curr Opin Crit Care* 2010;**16**:499–504. doi:10.1097/MCC.0b013e32833cb874

6 Stelfox HT, Lane D, Boyd JM, *et al.* A Scoping Review of Patient Discharge From Intensive Care. *Chest* 2015;**147**:317–27. doi:10.1378/chest.13-2965

7 Obermeyer Z, Lee TH. Lost in Thought — The Limits of the Human Mind and the Future of Medicine. *N Engl J Med* 2017;**377**:1209–11. doi:10.1056/NEJMp1705348

8 Docherty AB, Lone NI. Exploiting big data for critical care research. *Curr Opin Crit Care* 2015;**21**:467–72. doi:10.1097/MCC.0000000000000228

9 Sohn E, Roski J, Escaravage S, *et al.* Four Lessons In The Adoption Of Machine Learning In Health Care. *Heal Aff Blog* 2017. doi:10.1377/hblog20170509.059985

10 Kawamoto K, Houlihan CA, Balas EA, *et al.* Improving clinical practice using clinical decision support systems: a systematic review of trials to identify features critical to success. *BMJ* 2005;**330**:765. doi:10.1136/bmj.38398.500764.8F

11 Rojas JC, Carey KA, Edelson DP, *et al.* Predicting intensive care unit readmission with machine learning using electronic health record data. *Ann Am Thorac Soc* Published Online First: 2018. doi:10.1513/AnnalsATS.201710-787OC

12 Badawi O, Breslow MJ. Readmissions and Death after ICU Discharge: Development and Validation of Two Predictive Models. *PLoS One* Published Online First: 2012. doi:10.1371/journal.pone.0048758

13 Badawi O, Liu X, Hassan E, *et al.* Evaluation of ICU risk models adapted for use as continuous markers of severity of illness throughout the ICU stay. *Crit Care Med* Published Online First: 2018. doi:10.1097/CCM.0000000000002904

14 Cosgriff CV, Celi LA, Sauer CM. Boosting Clinical Decision-making: Machine Learning for Intensive Care Unit Discharge. *Ann Am Thorac Soc* 2018;**15**:804–5. doi:10.1513/AnnalsATS.201803-205ED

For peer review only

15 Desautels T, Das R, Calvert J, *et al.* Prediction of early unplanned intensive care unit readmission in a UK tertiary care hospital: A cross-sectional machine learning approach. *BMJ Open* Published Online First: 2017. doi:10.1136/bmjopen-2017-017199

16 Jeffs E, Vollam S, Young JD, *et al.* Wearable monitors for patients following discharge from an intensive care unit: practical lessons learnt from an observational study. *J Adv Nurs* 2016;**72**:1851–62. doi:10.1111/jan.12959

17 Escobar GJ, LaGuardia JC, Turk BJ, *et al.* Early detection of impending physiologic deterioration among patients who are not in intensive care: Development of predictive models using data from an automated electronic medical record. *J Hosp Med* 2012;**7**:388–95. doi:10.1002/jhm.1929

18 Harris S, Singer M, Rowan K, *et al.* Delay to admission to critical care and mortality among deteriorating ward patients in UK hospitals: a multicentre, prospective, observational cohort study. *Lancet* 2015;**385**:S40. doi:10.1016/S0140-6736(15)60355-5

19 Bourdeaux CP, Davies KJ, Thomas MJC, *et al.* Using 'nudge' principles for order set design: a before and after evaluation of an electronic prescribing template in critical care. *BMJ Qual Saf* 2014;**23**:382–8. doi:10.1136/bmjqs-2013-002395

20 Bourdeaux CP, Thomas MJ, Gould TH, *et al.* Increasing compliance with low tidal volume ventilation in the ICU with two nudge-based interventions: evaluation through intervention time-series analyses. *BMJ Open* 2016;**6**:e010129. doi:10.1136/bmjopen-2015-010129

21 Bourdeaux CP, Birnie K, Trickey A, *et al.* Evaluation of an intervention to reduce tidal volumes in ventilated ICU patients † ‡. *Br J Anaesth* 2015;**115**:244–51. doi:10.1093/bja/aev110

22 Knight G. Nurse-led discharge from high dependency unit. *Nurs Crit Care* 2003;**8**:56–61.

23 Johnson AEW, Pollard TJ, Shen L, *et al.* MIMIC-III, a freely accessible critical care database. *Sci Data* 2016;**3**:160035. doi:10.1038/sdata.2016.35

24 Crocker C, Keller R. Nurse-led discharge to the ward from high dependency: A service improvement project. *Intensive Crit Care Nurs* 2005;**21**:363–6. doi:10.1016/J.ICCN.2005.05.004

25 Lin F, Chaboyer W, Wallis M. A literature review of organisational, individual and teamwork factors contributing to the ICU discharge process. *Aust Crit Care* 2009;**22**:29–43. doi:10.1016/j.aucc.2008.11.001

26 Brown SES, Ratcliffe SJ, Halpern SD. An empirical derivation of the optimal time interval for defining ICU readmissions. *Med Care* 2013;**51**:706–14. doi:10.1097/MLR.0b013e318293c2fa

27 Vollam S, Dutton S, Lamb S, *et al.* Out-of-hours discharge from intensive care, in-hospital mortality and intensive care readmission rates: a systematic review and meta-analysis. *Intensive Care Med* 2018;**44**:1115–29. doi:10.1007/s00134-018-5245-2

For peer review only

28 Batista GEAPA, Monard MC. An analysis of four missing data treatment methods for supervised learning. *Appl Artif Intell* 2003;**17**:519–33. doi:10.1080/713827181

29 Maaten L van der, Hinton G. Visualizing Data using t-SNE. *J Mach Learn Res* 2008;**9**:2579–605.

30 Liaw A, Wiener M. Classification and Regression by RandomForest. *R News* 2002;**2**:18–22.

31 Dreiseitl S, Ohno-Machado L. Logistic regression and artificial neural network classification models: a methodology review. *J Biomed Inform*;**35**:352–9.

32 Geras KJ, Sutton C. Multiple-source cross-validation. Proc. 30th Int. Conf. Int. Conf. Mach. Learn. - Vol. 28. 2013;:III-1292.

33 Breiman L. Random Forests. *Mach Learn* 2001;**45**:5–32. doi:10.1023/A:1010933404324

34 Davis J, Goadrich M. The relationship between Precision-Recall and ROC curves. In: *Proceedings of the 23rd international conference on Machine learning - ICML '06*. New York, New York, USA: : ACM Press 2006. 233–40. doi:10.1145/1143844.1143874

35 Goldfrad C, Rowan K. Consequences of discharges from intensive care at night. *Lancet (London, England)* 2000;**355**:1138–42. doi:10.1016/S0140-6736(00)02062-6

36 Vranas KC, Jopling JK, Sweeney TE, *et al.* Identifying Distinct Subgroups of ICU Patients. *Crit Care Med* 2017;**45**:1607–15. doi:10.1097/CCM.0000000000002548

37 Yang J, Nguyen MN, San PP, *et al.* Deep Convolutional Neural Networks on Multichannel Time Series for Human Activity Recognition. *Twenty-Fourth Int Jt Conf Artif Intell* 2015.

38 Retter A, Wyncoll D, Pearse R, *et al.* Guidelines on the management of anaemia and red cell transfusion in adult critically ill patients. *Br J Haematol* 2013;**160**:445–64. doi:10.1111/bjh.12143

39 Alaa AM, Yoon J, Hu S, *et al.* Personalized Risk Scoring for Critical Care Prognosis Using Mixtures of Gaussian Processes. *IEEE Trans Biomed Eng* 2018;**65**:207–18. doi:10.1109/TBME.2017.2698602

40 van Buuren S. Multiple imputation of discrete and continuous data by fully conditional specification. *Stat Methods Med Res* 2007;**16**:219–42. doi:10.1177/0962280206074463

41 Lin J-H, Haug PJ. Exploiting missing clinical data in Bayesian network modeling for predicting medical problems. *J Biomed Inform* 2008;**41**:1–14. doi:10.1016/J.JBI.2007.06.001

42 Bakker J, Damen J, van Zanten ARH, *et al.* [Admission and discharge criteria for intensive care departments]. *Ned Tijdschr Geneeskd* 2003;**147**:110–5.

43 Hosein F, Bobrovitz N, Berthelot S, *et al.* A systematic review of tools for predicting severe adverse events following patient discharge from intensive care units. *Crit Care* 2013;**17**:R102. doi:10.1186/cc12747

44 Pollard TJ, Johnson AEW, Raffa JD, *et al.* The eICU Collaborative Research Database, a freely available multi-center database for critical care research. *Sci data* 2018;**5**:180178. doi:10.1038/sdata.2018.178

For peer review only

45 Harris S, Shi S, Brealey D, *et al.* Critical Care Health Informatics Collaborative (CCHIC): Data, tools and methods for reproducible research: A multi-centre UK intensive care database. *Int J Med Inform* 2018;**112**:82–9. doi:10.1016/j.ijmedinf.2018.01.006

46 Parker M. The Aggregation of Marginal Gains. *Bull R Coll Surg Engl* 2011;**93**:236–7. doi:10.1308/147363511X582239

47 Ribeiro MT, Singh S, Guestrin C. 'Why Should I Trust You?': Explaining the Predictions of Any Classifier. In: *Proceedings of the 22nd ACM SIGKDD International Conference on Knowledge Discovery and Data Mining - KDD '16*. New York, New York, USA: : ACM Press 2016. 1135–44. doi:10.1145/2939672.2939778

48 Deng H. Interpreting tree ensembles with inTrees. *Int J Data Sci Anal* 2018;:1–11. doi:10.1007/s41060-018-0144-8

49 Holzinger A. Interactive machine learning for health informatics: when do we need the human-in-the-loop? *Brain Informatics* 2016;**3**:119–31. doi:10.1007/s40708-016-0042-6

For peer review only

Figure legends:

Figure 1: Performance of the nurse-led discharge criteria (NLD) and random forest with extended feature set (RF_{ext}) evaluated on held-out data for a single train-test split. Left: ROC curves with associated area-under-curve (AUC) scores. Right: precision-recall curves.

For peer review only

Contributors:

CJM is the main author and conducted the data processing and analysis. RSR and DJL made important technical and methodological contributions. IDG, AC, CPB drove the study concept and design. The clinical expertise of THG, MJCT and CPB informed all stages of the project, in particular study design and interpretation of results. All authors contributed to writing the manuscript and approved the final version.

Funding:

CJM was funded by the Elizabeth Blackwell Institute Catalyst Fund. DJL is funded by Wellcome Trust and Royal Society Grant Number WT104125MA.

For peer review only

Acknowledgements:

We would like to thank Graeme Palmer and Amy Weaver for their support in accessing and understanding the GICU data.

Competing interests:

None declared.

Ethics approval:

Not required. CAG guidelines followed and study protocol presented to University Hospitals Bristol NHS Foundation Trust institutional review board.

Patient and Public Involvement :

Patients were not directly involved in this study and patient consent was not required.

Data sharing statement:

Feature matrices will be made available on Dryad. Python code for analysis and processing on request directly from the corresponding author.

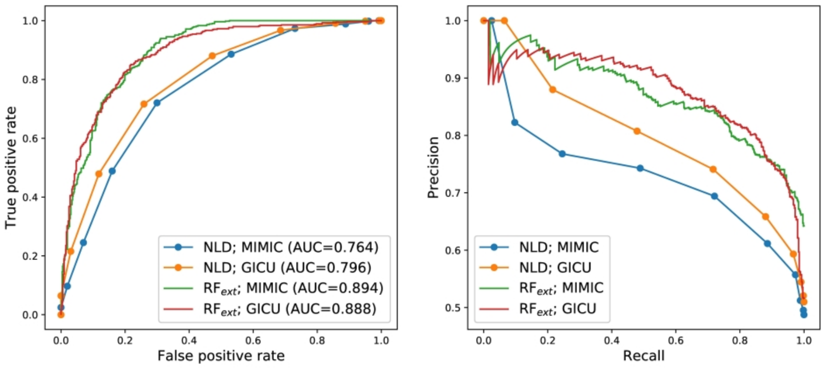

Figure 1: Performance of the nurse-led discharge criteria (NLD) and random forest with extended feature set (RFext) evaluated on held-out data for a single train-test split. Left: ROC curves with associated area-under-curve (AUC) scores. Right: precision-recall curves.

355x152mm (300 x 300 DPI)

Supplementary online file for: A machine learning approach to intensive care discharge.

Correspondance to: chris.mcwilliams@bristol.ac.uk

Contents

A Codifying discharge criteria **2**

B Data preprocessing **3**

B.1 Data harmonisation and concept mapping 3

B.2 Inclusion criteria 4

B.3 Time of day effects 7

C Feature extraction and missing data **8**

D Complete case analysis **10**

E Implementation in code **16**

For peer review only - http://bmjopen.bmj.com/site/about/guidelines.xhtml

A Codifying discharge criteria

The original specification of the nurse-led discharge criteria [1] is presented as a check list with some room for user interpretation. For example, one of the check list questions is: *'Urea and electrolytes normal?'* In order to apply the NLD criteria to historical data we needed to codify them and remove any ambiguity. To do so we first split the original criteria into 16 binary tests, where each test places an upper and/or lower bound on a single physiological variable. If the variable value lies within the bounds the test is passed. For the *'Urea and electrolytes'* criterion we defined four separate tests: one for urea and for each of the three electrolytes identified, via clinical consultation, as most relevant to patient discharge (potassium, sodium, creatinine). We took the 'normal' values of these four variables from the bounds specified in the Philips DAR system in use on GICU.

The blood gas test (R2) was originally multi-variable, either testing the arterial blood gases (PaO2, PaCO2) or the *peripheral capillary oxygen saturation* (SpO2) in the absence of an arterial line. To simplify R2 we neglected PaO2 and PaCO2 (which were missing with high frequency), and tested only SpO2 for all patients.

The airway test (R0) checks if the patient has a patent airway. For this test we used the absence of an endotracheal tube (ETT) as a proxy for the airway being patent.

Finally, we neglected the test of urine output since this could not be successfully located in MIMIC (see section B.1).

Following this procedure we obtained the 15 codified binary tests presented in table 1 of the main text, and referred to throughout as the NLD criteria.

We note here that the criterion value of $9g/dL$, above which haemoglobin is deemed acceptable, does not appear to be evidence based. We refer the

For peer review only - http://bmjopen.bmj.com/site/about/guidelines.xhtml

reader to this discussion [2] on haemoglobin in critical care. More generally this highlights the problematic nature of scoring systems that place fixed boundary constraints on physiological parameters, even when these boundary values are evidence based. We feel that this motivates the power and algorithmic flexibility of a machine learning approach.

B Data preprocessing

In this section of the supplementary information we cover the details of the data preprocessing that was required prior to analysis. The basics of these procedures are outlined in the main text, and referenced to the relevant part of this section fuller for explanation.

B.1 Data harmonisation and concept mapping

Extensive pre-processing was required for both MIMIC and GICU in order to put the data in a workable format. One reason for this is that both datasets are derived from clinical information systems (CIS) that were never designed for secondary research purposes. Both MIMIC and GICU datasets contained problems with inconsistent naming conventions for physiological parameters. We determined that this problem was less severe in the portion of MIMIC-III derived from the *Metavision* data source (as compared to *Physionet*). Therefore, we restricted our study to this subset of the data. Despite this, extensive concept mapping of disparate *interventionIDs* was required in order to obtain physiological records that were coherent and as close to complete as possible. It was not possible to identify a coherent measure of urine output in MIMIC-III. This is an issue that has previously been discussed on the forums accompanying the MIMIC website. Therefore, we were forced to neglect the discharge criteria based on urine output throughout this study. In general the GICU dataset was

For peer review only - http://bmjopen.bmj.com/site/about/guidelines.xhtml

easier to work with, in no small part thanks to working with the clinicians who are responsible for administering the associated CIS. When working with both datasets particular care was taken to ensure consistency of measurement units, which differ between the two systems.

B.2 Inclusion criteria

As described in the main text we selected study subjects from the MIMIC and GICU datasets according to a number of inclusion criteria. The main goal was to obtain approximately equal sized samples of both patients who were ready for discharge, and those who were not ready for discharge. The GICU data is derived from the clinical information system in use on the general intensive care unit at the Bristol Royal Infirmary. GICU is a combined critical care and high-dependency unit that treats both level 2 and level 3 patients. The MIMIC data is derived from patients admitted to critical care units at the Beth Israel Deaconess Medical Center in Boston, Massachusetts between 2001 and 2012. Initially we applied the following criteria when selecting subjects from the two historical datasets:

- From MIMIC we selected patients from MIMIC-III who were admitted to either medical or surgical intensive care, since this approximates the patient type admitted to general intensive care in Bristol.
- From MIMIC we selected only patient from *Metavision* (see section B.1).
- We only included the first intensive care stay of any given hospital admission. This decision allowed us to calculate readmission rates to ICU. (Readmission was one negative outcome considered following callout.)
- From GICU we included only patients who could be linked to Ward-Watcher (administrative system) via their lifetime ID number.

For peer review only - http://bmjopen.bmj.com/site/about/guidelines.xhtml

- From both data sources we selected only patients with a recorded callout (ready for discharge time).
- Furthermore, we discarded any patients (GICU and MIMIC) that had three of more feature variables with no recorded value.
- The two resulting cohorts of study subjects are summarised in table 2 of the main text.

The first step in processing the data was to omit those intensive care stays with no recorded callout. This omission removed any patients who died on ICU, as well as those who should have had a callout recorded but for some reason it was missing in the data. The second step was to define positive and negative outcomes following callout (see main text). Patients with a positive outcome were determined to be ready for discharge at the time of callout. Patients with a negative outcome were determined to be not ready for discharge, either because they were erroneously discharged or because they were discharged in the knowledge that they were not fit (e.g. specialist unit transfer or palliative). For MIMIC and GICU the rate of negative outcomes was 12.57% and 5.83% percent respectively (see the bars at $X_i = 0$ in figure 1).

The next step was to increase the number of instances of NRFD in order to balance to class sizes for training and testing purposes. To produce more instances of NRFD we sampled from earlier in patient stays at times when they were assumed to be not clinically fit for discharge. Heuristically we made the decision that we should sample at least three days prior to callout to ensure no ambiguity in RFD status (one or two days prior to callout it may be the case that patients were RFD but were being held on to conservatively). We also chose to exclude patients who were in the first 24 hours of their ICU stay from this sampling. From figure 1 it is clear that, given the distribution in the length of patient stays, there is only so far back in time we can sample and still obtain

For peer review only - http://bmjopen.bmj.com/site/about/guidelines.xhtml

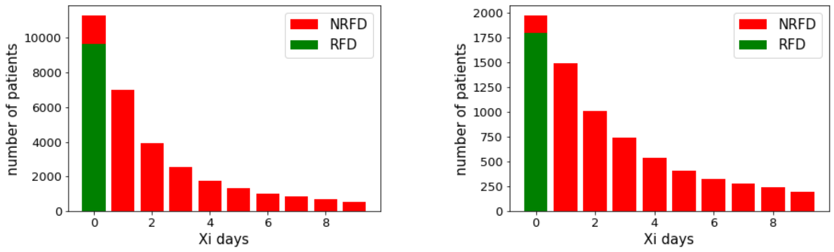

MIMIC *GICU*

Figure 1: *Illustrating instances sampled at in integer multiples (X_i) of 24 hours prior to callout. Patient callout is at $X_i = 0$. At this time patients with positive outcome are considered ready for discharge (RFD), and patients with negative outcome are considered not ready for discharge (NRFD). At $X_i > 0$ all patients are considered NRFD regardless of subsequent outcome status.*

enough instances of NRFD to balance the class sizes. Therefore, we sampled at time points from to three to eight days prior to callout ($X_i = [3, 4, 5, 6, 7, 8]$). The reason for sampling in exact multiples of 24 hours prior to callout was to ensure the removal of time of day effects from the data (see section B.3). An alternative strategy would be to sample and classify patients at a set time point each day. This strategy would have the benefit of preserving the time relationship of certain daily activities. For example, daily bloods are likely done in the morning. Our strategy, of sampling in 24 hour multiples prior to callout may introduce more variability in the time since last bloods, and possibly other measurements/interventions. However, given that we wanted to work towards a classifier that is not constrained by activities (such as discharge decisions) occurring at fixed times of day, we feel that the chosen sampling strategy is justified.

Sampling patient stays at $X_i = [3, 4, 5, 6, 7, 8]$ prior to callout yielded 8425 and 3174 instances for MIMIC and GICU respectively. We then combined these extra NRFD instances with the original instances (RFD and NRFD) sampled at time of callout ($X_i = 0$), and re-sampled the larger class to obtain a 50:50

For peer review only

For peer review only - http://bmjopen.bmj.com/site/about/guidelines.xhtml

ratio of RFD:NRFD. This re-sampling was conducted with the Python method: *SFrame.sample(fraction,seed)*. The resulting datasets for MIMIC and GICU contained 7592 and 1870 instances respectively. The subsequent removal of instances with missing data (see section C) reduced these to 5038 and 1851.

B.3 Time of day effects

Discharge decisions are invariability made in the morning (figure 2) after discussion at the morning hand over meeting and subsequent ward round. There are non-random temporal signals in the value of certain physiological variables (figure 3). Therefore, it is possible to train a reasonable classifier to identify readiness for discharge by recognising non-random variation in variable values due to time of day. For example: blood pressure is lower than average → it must be morning time → this patient is more likely to be RFD. This highlights a problem in identifying when patients are actually physically fit for discharge, versus when they are declared so. It also means we must be careful when introducing extra instances of the negative class by sampling from earlier in patient stays - this is why we always sampled in integer multiples of 24 hours prior to callout.

The frequency with which the callout decision is taken around 11am (figure 2) highlights one important way that a decision support system could improve upon ICU discharge practice: by prompting clinicians to consider discharge at other, non-specified time points during the day.

For peer review only - http://bmjopen.bmj.com/site/about/guidelines.xhtml

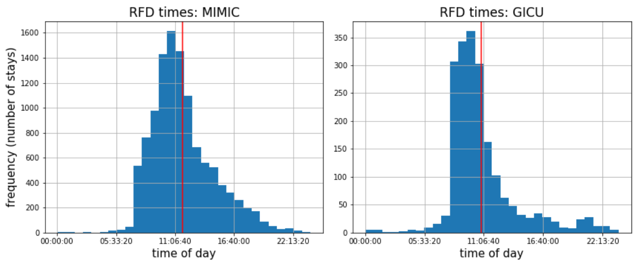

Figure 2: *Histogram of the time of day at which patients were declared ready for discharge (callout time) for: MIMIC (left) and GICU (right). The red line indicates the mean of the distribution.*

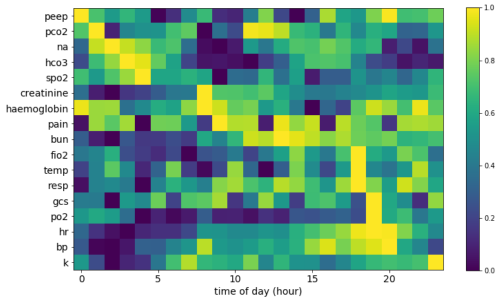

Figure 3: *Variation in average physiological variable values by time of day for the GICU dataset. We calculated the average value for a number of physiological variables at each hour of the day over all patients in the GICU dataset. The values for each variable are than scaled between 0 and 1 to be displayed on the heatmap. Certain temporal patterns become visible. For example, heart rate (HR) and blood pressure (BP) tend to be higher in the evening.*

C Feature extraction and missing data

We constructed feature matrices for each cohort such that the NLD criteria could easily be tested, and machine learning classifiers could be trained on the same data. In these feature matrices each row represents an instance of either RFD of NRFD, and each column represents a feature. Each of the NLD criteria tests the numeric value of a single physiological parameter. Certain criteria test whether the parameter lies within specified lower and upper bounds, while others impose

For peer review only - http://bmjopen.bmj.com/site/about/guidelines.xhtml

only a single (lower or upper) bound (see table 1). Therefore, to evaluate a criterion with two bounds requires two feature columns: one giving the minimum value of the relevant parameter, and the other giving the maximum. A single bound criterion requires only one feature column. For example, the features 'resp_min' and 'resp_max' are required for test R4, whereas to test R1 the feature 'fio2_max' alone is sufficient.

We calculated the required minima and maxima of the physiological parameters over four-hour sample windows, as specified by the original NLD criteria. For the original instances (RFD and NRFD) this window constituted the four hours immediately preceding callout. For the extra NRFD instances this was the four hour window immediately preceding the earlier sample time (X_i days prior to callout). In both cases the resulting feature columns contained large amounts of missing data where variables had not been recorded during the specified window, as shown in table 1. This problem was worse with those variables derived from laboratory tests (such as 'bun'), which are infrequently collected (approximately once every 24 hours). To replace some of the missing values the sample window was extended from four to 36 hours. If a missing variable was recorded during this extended window, the latest recorded value was taken for both the minimum and maximum. Clinically it was expected that the main source of missing data is that a variable is less likely to be recorded if it is not of clinical concern. In such cases the extended sample window represents a conservative estimation of a patient's physiological state, by taking parameter values at the last time when they were of concern to clinicians. For the five variables corresponding to double-bound NLD criteria, but with large amounts of missing data (pain, k, bun, creatinine, na) the decision was taken to produce only a single feature for each, rather than two, by taking only the final recorded value. This reduction avoided significant value duplication between feature columns.

For peer review only - http://bmjopen.bmj.com/site/about/guidelines.xhtml

Variables	MIMIC		GICU	
	at CALLOUT	Xi days prior to CALLOUT	at CALLOUT	Xi days prior to CALLOUT
hr_max	0.002 (0.000)	0.007 (0.005)	0.018 (0.003)	0.195 (0.194)
hr_min	0.002 (0.000)	0.007 (0.005)	0.018 (0.003)	0.195 (0.194)
spo2_max	0.011 (0.001)	0.009 (0.005)	0.027 (0.004)	0.202 (0.195)
spo2_min	0.011 (0.001)	0.009 (0.005)	0.027 (0.003)	0.202 (0.195)
airway	0.985 (0.000)	0.511 (0.000)	0.057 (0.000)	0.215 (0.000)
resp_max	0.006 (0.001)	0.009 (0.006)	0.057 (0.007)	0.203 (0.195)
resp_min	0.006 (0.001)	0.009 (0.006)	0.057 (0.007)	0.203 (0.195)
bp_min	0.007 (0.000)	0.008 (0.005)	0.069 (0.039)	0.254 (0.235)
bp_max	0.007 (0.000)	0.008 (0.005)	0.069 (0.039)	0.254 (0.235)
temp_min	0.077 (0.002)	0.079 (0.026)	0.113 (0.004)	0.229 (0.197)
temp_max	0.077 (0.002)	0.079 (0.026)	0.113 (0.004)	0.229 (0.197)
gcs_max	0.106 (0.002)	0.086 (0.011)	0.0121 (0.005)	0.233 (0.196)
gcs_min	0.106 (0.002)	0.086 (0.011)	0.0121 (0.005)	0.233 (0.196)
pain	0.461 (0.140)	0.466 (0.152)	0.194 (0.033)	0.507 (0.372)
hco3	0.885 (0.019)	0.847 (0.037)	0.490 (0.075)	0.312 (0.152)
fio2	0.932 (0.686)	0.380 (0.264)	0.767 (0.402)	0.455 (0.320)
haemoglobin	0.894 (0.024)	0.878 (0.042)	0.975 (0.016)	0.803 (0.026)
k	0.875 (0.017)	0.810 (0.034)	0.975 (0.014)	0.805 (0.028)
bun	0.884 (0.019)	0.854 (0.038)	0.975 (0.013)	0.803 (0.025)
creatinine	0.884 (0.019)	0.854 (0.037)	0.975 (0.015)	0.805 (0.027)
na	0.875 (0.017)	0.822 (0.034)	0.975 (0.013)	0.803 (0.025)

Table 1: *Summary of missing data in the MIMIC and GICU feature matrices. Values are provided for the samples drawn at time of callout, and samples drawn prior to callout (at* X_i*=[3,4,5,6,7,8]). The main values are the fraction of instances with no value recorded during the four hour window. Values in brackets give fraction of missing data when sample window is extended to 36 hours (see text).*

D Complete case analysis

In medical data there is likely to be meaning in missingness [3] i.e. data is not missing at random but often due to some other reason. For historical data it is not easy to discern the reasons for missing data, but a naive treatment of missingness is liable to bias the dataset and also to lose valuable information from which to do statistical learning. In the main text we presented results produced from an imputed version of the dataset. To impute the values of missing data entries we used k-nearest neighbours imputation (with $k = 5$). To do this a k-nearest neighbours model was constructed from the complete cases (instances with no missing data). This model was then queried and the mean feature value of the $k = 5$ nearest-neighbours of each instance was taken to replace any missing feature values for that instance. In this section we reproduce the results from the main text but using a complete case analysis with all patients with

For peer review only - http://bmjopen.bmj.com/site/about/guidelines.xhtml

missing data entries removed. In this way we investigate the sensitivity our results to the missing data values and the k-nn imputation strategy. Beyond this, the optimal treatment of missing data represents a significant avenue for development prior to the deployment of our methodology as a real-time decision support tool.

The removal of the entries with missing data (i.e. complete case analysis) does not change the following key results:

- The random forest and logistic classifiers perform better than the original NLD criteria.
- Weighting the NLD criteria by the feature importance of the logistic classifier improves performance.
- Broadly, which features are most predictive of readiness for discharge remains unchanged, and is consistent between classifiers (Spearman's rank correlation coefficient 0.800 here, 0.761 for imputed data set).

The main difference introduced when moving from the complete case analysis to the imputed data set is slight a drop in performance across the board for the MIMIC cohort. On aggregate it becomes harder to identify MIMIC patients that are ready for discharge when those with missing entries are included. For GICU there is a slight change in the opposite direction. The NLD, random forest and logistic classifier all perform slightly better when GICU patients with missing data entries are included. These performance changes suggest non-random structures in the way that data entries are missing, which likely relate to differences in clinical practice. For example it may be that MIMIC patients that are *nearly* ready for discharge a monitored less closely than more sick patients and therefore have more missing data. The inclusion of patients with missing data would introduce more of these ambiguous cases, which are nearly

For peer review only - http://bmjopen.bmj.com/site/about/guidelines.xhtml

	Predicted RFD	Predicted NRFD
RFD	11	2496
NRFD	2	2529

Table 2: *Confusion matrix for NLD criteria applied to MIMIC feature matrix, with missing data entries removed (complete case analysis).*

	Predicted RFD	Predicted NRFD
RFD	60	871
NRFD	5	915

Table 3: *Confusion matrix for NLD criteria applied to GICU feature matrix, with missing data entries removed (complete case analysis).*

	Predicted RFD	Predicted NRFD
RFD	75	6563
NRFD	8	6597

Table 4: *Confusion matrix for NLD criteria applied to MIMIC feature matrix, with missing data filled using k-nearest neighbours. Equivalent to table 2.*

	Predicted RFD	Predicted NRFD
RFD	117	1644
NRFD	7	1740

Table 5: *Confusion matrix for NLD criteria applied to GICU feature matrix, with missing data filled using k-nearest neighbours. Equivalent to table 3.*

RFD but still being held in critical care. Although the performance changes do not alter the conclusions drawn from the results in the main text, they do suggest that strategies for dealing with missing data should be developed in accordance with on-site clinical practice when deploying such a tool in order to achieve optimal classifications.

For peer review only

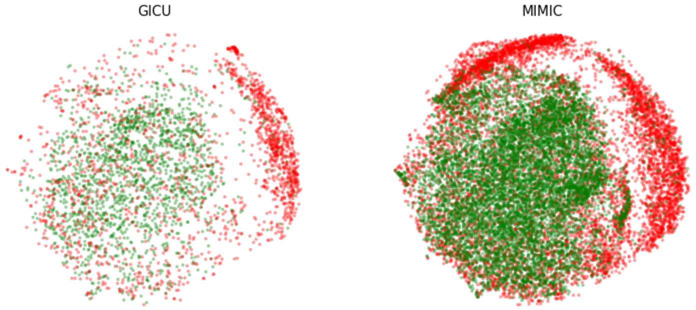

Figure 4: *t-SNE embedding [4] of the feature space for GICU (left), and MIMIC (right), for the imputed dataset. Green and red points indicate instances of RFD and NRFD respectively. The more similar two instances (in terms of feature values), the closer together they appear in the embedding space.*

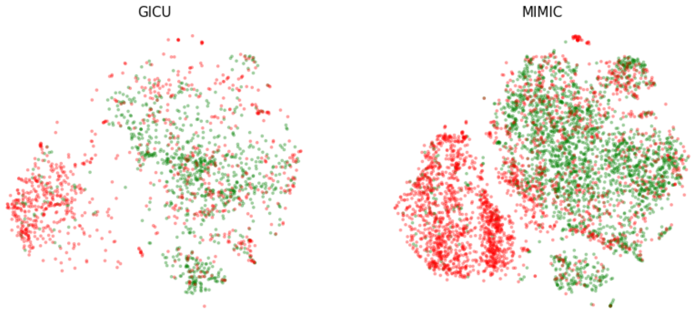

Figure 5: *Equivalent to figure 4, but for the complete case data set.*

For peer review only - http://bmjopen.bmj.com/site/about/guidelines.xhtml

		NLD	NLD_weighted	LC	RF	LC_extended	RF_extended
GICU	AUROC	0.7802 (0.0173)	0.8058 (0.0170)	0.8680 (0.0142)	0.8544 (0.0148)	**0.8730 (0.0139)**	0.8619 (0.0146)
	Accuracy	0.7110 (0.0297)	0.7582 (0.0355)	**0.8284 (0.0501)**	0.8215 (0.0471)	0.8279 (0.0485)	0.8198 (0.0479)
	F1	0.7366 (0.0176)	0.7543 (0.0241)	0.8007 (0.0145)	0.7966 (0.0166)	**0.8010 (0.0144)**	0.7954 (0.0184)
	Specificity	0.7000 (0.0000)	0.7000 (0.0000)	0.7000 (0.0000)	0.7000 (0.0000)	0.7000 (0.0000)	0.7000 (0.0000)
	pAUROC	0.1412 (0.0096)	0.1426 (0.0120)	0.1920 (0.0099)	0.1815 (0.0103)	**0.1951 (0.0098)**	0.1853 (0.0106)
	Brier	0.2694 (0.0085)	0.2419 (0.0124)	0.1598 (0.0085)	0.1675 (0.0076)	**0.1578 (0.0085)**	0.1623 (0.0080)
	Sensitivity	0.7302 (0.0281)	0.7750 (0.0405)	**0.8750 (0.0232)**	0.8640 (0.0255)	0.8732 (0.0218)	0.8615 (0.0298)
MIMIC	AUROC	0.7544 (0.0097)	0.8686 (0.0076)	0.8880 (0.0137)	0.8898 (0.0143)	0.8910 (0.0131)	**0.8990 (0.0125)**
	Accuracy	0.6747 (0.0148)	0.8545 (0.0537)	0.8641 (0.0607)	0.8651 (0.0617)	0.8646 (0.0609)	**0.8775 (0.0654)**
	F1	0.7196 (0.0099)	0.8203 (0.0091)	0.8248 (0.0155)	0.8257 (0.0170)	0.8254 (0.0153)	**0.8339 (0.0141)**
	Specificity	0.7000 (0.0000)	0.7000 (0.0000)	0.7000 (0.0000)	0.7000 (0.0000)	0.7000 (0.0000)	0.7000 (0.0000)
	pAUROC	0.1315 (0.0052)	0.1816 (0.0064)	0.1979 (0.0117)	0.1987 (0.0122)	0.2017 (0.0109)	**0.2047 (0.0114)**
	Brier	0.2443 (0.0039)	0.1551 (0.0068)	0.1319 (0.0082)	0.1343 (0.0074)	0.1303 (0.0078)	**0.1279 (0.0066)**
	Sensitivity	0.6724 (0.0184)	0.9070 (0.0145)	0.9221 (0.0224)	0.9230 (0.0268)	0.9225 (0.0234)	**0.9405 (0.0217)**

Table 6: *Performance summary for the complete case analysis, equivalent to table 3 in the main text. All scores are averaged over 100 train-test data splits and given as: mean (standard deviation). All metrics other than AUROC and Brier score are evaluated at a specificity of 0.7, using linear interpolation to estimate this operating point in ROC-space.* $NLD_{weighted}$ *are the NLD criteria, weighted by feature importances from the logistic classifier (table 4).* $LC_{extended}$ *and* $RF_{extended}$ *are the machine learning classifiers with extended feature sets.*

	FPR	NLD	NLD_weighted	LR	RF	LR_extended	RF_extended
GICU	0.1	0.4231 (0.0330)	0.4259 (0.0341)	0.6002 (0.0367)	0.5600 (0.0375)	**0.6274 (0.0327)**	0.5755 (0.0406)
	0.2	0.6109 (0.0234)	0.6334 (0.0256)	**0.7937 (0.0211)**	0.7633 (0.0308)	0.7915 (0.0221)	0.7744 (0.0283)
	0.3	0.7426 (0.0166)	0.8098 (0.0263)	0.8870 (0.0171)	0.8860 (0.0196)	0.8767 (0.0196)	**0.8909 (0.0185)**
	0.4	0.8204 (0.0152)	0.9244 (0.0125)	0.9417 (0.0104)	0.9418 (0.0093)	0.9404 (0.0123)	**0.9473 (0.0108)**
	0.5	0.8884 (0.0114)	0.9514 (0.0083)	0.9706 (0.0073)	0.9653 (0.0072)	**0.9766 (0.0074)**	0.9711 (0.0068)
	0.6	0.9253 (0.0092)	0.9752 (0.0050)	0.9860 (0.0047)	0.9815 (0.0061)	**0.9877 (0.0042)**	0.9824 (0.0057)
	0.7	0.9609 (0.0081)	0.9804 (0.0047)	0.9903 (0.0041)	0.9886 (0.0046)	**0.9918 (0.0037)**	0.9897 (0.0044)
	0.8	0.9791 (0.0047)	0.9899 (0.0036)	0.9945 (0.0030)	0.9929 (0.0035)	**0.9951 (0.0028)**	0.9937 (0.0034)
	0.9	0.9927 (0.0025)	0.9973 (0.0017)	0.9957 (0.0025)	0.9965 (0.0023)	0.9963 (0.0024)	**0.9970 (0.0022)**
	1	1.0000 (0.0000)	1.0000 (0.0000)	0.9999 (0.0004)	1.0000 (0.0000)	1.0000 (0.0000)	1.0000 (0.0002)
MIMIC	0.1	0.3370 (0.0128)	0.3740 (0.0185)	0.4611 (0.0484)	0.4858 (0.0454)	0.5449 (0.0482)	**0.5933 (0.0452)**
	0.2	0.5296 (0.0110)	0.6355 (0.0211)	0.7466 (0.0404)	0.7465 (0.0442)	0.7825 (0.0304)	**0.7881 (0.0349)**
	0.3	0.6713 (0.0126)	0.8337 (0.0174)	0.8827 (0.0282)	0.8860 (0.0265)	0.9001 (0.0207)	**0.9049 (0.0210)**
	0.4	0.7545 (0.0086)	0.9295 (0.0091)	0.9526 (0.0152)	0.9498 (0.0158)	0.9590 (0.0111)	**0.9597 (0.0125)**
	0.5	0.8309 (0.0096)	0.9777 (0.0045)	0.9842 (0.0075)	0.9853 (0.0065)	0.9872 (0.0060)	**0.9903 (0.0055)**
	0.6	0.8869 (0.0069)	0.9925 (0.0016)	0.9943 (0.0035)	0.9952 (0.0030)	0.9939 (0.0031)	**0.9973 (0.0026)**
	0.7	0.9315 (0.0056)	0.9950 (0.0013)	0.9961 (0.0028)	0.9972 (0.0024)	0.9961 (0.0024)	**0.9985 (0.0018)**
	0.8	0.9659 (0.0036)	0.9969 (0.0010)	0.9980 (0.0019)	0.9985 (0.0018)	0.9971 (0.0022)	**0.9991 (0.0015)**
	0.9	0.9887 (0.0022)	0.9990 (0.0006)	0.9993 (0.0011)	0.9996 (0.0008)	0.9982 (0.0019)	**0.9997 (0.0007)**
	1	1.0000 (0.0000)	1.0000 (0.0000)	1.0000 (0.0000)	1.0000 (0.0000)	0.9998 (0.0005)	1.0000 (0.0000)

Table 7: *Sensitivity values for the various classifiers over a range of false positive rates (FPR), for the* ***imputed data set****. Specificity = 1 - FPR.*

For peer review only - http://bmjopen.bmj.com/site/about/guidelines.xhtml

	FPR	NLD	$NLD_{weighted}$	LR	RF	$LR_{extended}$	$RF_{extended}$
GICU	0.1	0.4003 (0.0433)	0.4117 (0.0564)	0.5944 (0.0413)	0.5405 (0.0521)	**0.6142 (0.0431)**	0.5733 (0.0490)
	0.2	0.5928 (0.0389)	0.6074 (0.0385)	0.7731 (0.0397)	0.7470 (0.0414)	**0.7815 (0.0367)**	0.7492 (0.0400)
	0.3	0.7302 (0.0281)	0.7750 (0.0405)	**0.8750 (0.0232)**	0.8640 (0.0255)	0.8732 (0.0218)	0.8615 (0.0298)
	0.4	0.8053 (0.0252)	0.8899 (0.0241)	0.9188 (0.0201)	0.9143 (0.0193)	0.9222 (0.0199)	**0.9250 (0.0194)**
	0.5	0.8735 (0.0213)	0.9318 (0.0155)	0.9537 (0.0148)	0.9478 (0.0146)	**0.9589 (0.0141)**	0.9589 (0.0148)
	0.6	0.9164 (0.0158)	0.9612 (0.0132)	0.9734 (0.0110)	0.9631 (0.0113)	**0.9779 (0.0092)**	0.9726 (0.0101)
	0.7	0.9564 (0.0131)	0.9744 (0.0086)	0.9837 (0.0072)	0.9802 (0.0096)	**0.9868 (0.0066)**	0.9844 (0.0076)
	0.8	0.9764 (0.0077)	0.9846 (0.0060)	0.9925 (0.0051)	0.9885 (0.0064)	**0.9932 (0.0049)**	0.9907 (0.0049)
	0.9	0.9916 (0.0041)	0.9952 (0.0032)	0.9973 (0.0026)	0.9965 (0.0034)	**0.9974 (0.0024)**	0.9958 (0.0037)
	1	1.0000 (0.0000)	1.0000 (0.0000)	1.0000 (0.0000)	1.0000 (0.0000)	1.0000 (0.0000)	1.0000 (0.0000)
MIMIC	0.1	0.3880 (0.0238)	0.4841 (0.0392)	0.6001 (0.0603)	0.6030 (0.0651)	0.6211 (0.0598)	**0.6360 (0.0573)**
	0.2	0.5473 (0.0208)	0.7946 (0.0277)	0.8285 (0.0370)	0.8217 (0.0402)	0.8408 (0.0377)	**0.8417 (0.0393)**
	0.3	0.6724 (0.0184)	0.9070 (0.0145)	0.9221 (0.0224)	0.9230 (0.0268)	0.9225 (0.0234)	**0.9405 (0.0217)**
	0.4	0.7630 (0.0160)	0.9635 (0.0085)	0.9700 (0.0119)	0.9710 (0.0123)	0.9710 (0.0118)	**0.9819 (0.0107)**
	0.5	0.8433 (0.0117)	0.9794 (0.0044)	0.9849 (0.0072)	0.9884 (0.0058)	0.9825 (0.0075)	**0.9937 (0.0051)**
	0.6	0.8905 (0.0091)	0.9857 (0.0033)	0.9905 (0.0054)	0.9924 (0.0046)	0.9889 (0.0059)	**0.9962 (0.0036)**
	0.7	0.9373 (0.0086)	0.9901 (0.0032)	0.9935 (0.0041)	0.9939 (0.0044)	0.9914 (0.0051)	**0.9975 (0.0029)**
	0.8	0.9655 (0.0053)	0.9947 (0.0022)	0.9969 (0.0032)	0.9975 (0.0030)	0.9946 (0.0042)	**0.9987 (0.0019)**
	0.9	0.9872 (0.0032)	0.9978 (0.0015)	0.9987 (0.0019)	0.9995 (0.0013)	0.9977 (0.0025)	**0.9997 (0.0010)**
	1	1.0000 (0.0000)	1.0000 (0.0000)	1.0000 (0.0000)	1.0000 (0.0000)	0.9997 (0.0010)	1.0000 (0.0000)

Table 8: *Sensitivity values for the various classifiers over a range of false positive rates (FPR), for the* ***complete case data set****. Specificity = 1 - FPR.*

	Importance (LC)	Importance (RF)	Rank (LC)	Rank (RF)
**gcs_min**	0.1002 (0.0045)	0.0920 (0.0121)	0	0
**airway**	0.0874 (0.0105)	0.0780 (0.0117)	1	1
**bun**	0.0169 (0.0015)	0.0106 (0.0018)	2	3
**fio2**	0.0097 (0.0010)	0.0152 (0.0019)	3	2
**haemoglobin**	0.0090 (0.0011)	0.0093 (0.0018)	4	4
**hr_max**	0.0045 (0.0020)	0.0053 (0.0012)	5	5
**resp_max**	0.0040 (0.0016)	0.0017 (0.0005)	6	10
**hr_min**	0.0038 (0.0015)	0.0031 (0.0008)	7	6
**resp_min**	0.0025 (0.0015)	0.0031 (0.0006)	8	7
**spo2_min**	0.0012 (0.0007)	0.0005 (0.0002)	9	12
**hco3**	0.0004 (0.0003)	0.0002 (0.0001)	10	16
**temp_max**	0.0002 (0.0006)	0.0023 (0.0006)	11	8
**na**	0.0002 (0.0002)	0.0002 (0.0001)	12	15
**pain**	0.0001 (0.0001)	0.0010 (0.0003)	13	11
**temp_min**	0.0001 (0.0005)	0.0003 (0.0002)	14	14
**bp_min**	0.0001 (0.0001)	0.0005 (0.0002)	15	13
**k**	0.0000 (0.0000)	0.0002 (0.0001)	16	17
**creatinine**	0.0000 (0.0000)	0.0018 (0.0005)	17	9

Table 9: *Feature importances under the complete case analysis, equivalent to table 4 in the main text.*

E Implementation in code

Data extraction and preprocessing were conducted using SQL. We worked with a local MySQL instance of the MIMIC-III data, and the Bristol (GICU) data was accessed from the backend of the Philips DAR clinical information system via Microsoft SQL Reporting Services on the hospital server. Additional patient demographic information was obtained from the WardWatcher system, which is the ICNARC reporting software in use on the general intensive care unit in Bristol. Entries from WardWatcher were linked to the Philips data via patient lifetime identification numbers. The data were then anonymised by the removal of all sensitive information, including times and date, except for those variables required for the analysis (see main text). Subsequent data processing and analysis was conducted in Python within UH Bristol. We used the machine learning package scikit-learn to train and test classifiers. In particular the method *GridsearchCV* was used to optimise model hyperparamaters via multiple-source cross-validation, optimising for the F1-score. This optimisation was conducted over two folds, where each fold contained only data from a single source (either GICU or MIMIC). The 'best_estimator' parameter of *GridsearchCV* was set to 'True' such that the optimised model was refitted to the full training data (MIMIC and GICU). For the logistic classifier the regularisation hyperparameter 'c' was optimised over a logarithmic range of twenty values between 1×10^{-3} and 1×10^{3}. For the random forest three hyperparameters were optimised over the following ranges: $n\_estimators \in [20, 50, 100]$; $max\_features \in [20, 50, 100]$; $max\_depth \in [4, 5, 6, 7]$. Feature importances were calculated using the *PermutationImportance* method from the package ELI5, whereby the importance is taken as the average loss in performance (AUROC score) when the feature values are randomly permuted.

For peer review only - http://bmjopen.bmj.com/site/about/guidelines.xhtml

References

[1] Gillian Knight. Nurse-led discharge from high dependency unit. *Nursing in critical care*, 8(2):56–61, 2003.

[2] Andrew Retter, Duncan Wyncoll, Rupert Pearse, Damien Carson, Stuart McKechnie, Simon Stanworth, Shubha Allard, Dafydd Thomas, Tim Walsh, and British Committee for Standards in Haematology. Guidelines on the management of anaemia and red cell transfusion in adult critically ill patients. *British journal of haematology*, 160(4):445–464, 2013.

[3] Jau-Huei Lin and Peter J Haug. Exploiting missing clinical data in bayesian network modeling for predicting medical problems. *Journal of biomedical informatics*, 41(1):1–14, 2008.

[4] Laurens van der Maaten and Geoffrey Hinton. Visualizing data using t-sne. *Journal of Machine Learning Research*, 9(Nov):2579–2605, 2008.

For peer review only

For peer review only - http://bmjopen.bmj.com/site/about/guidelines.xhtml
